# Spatial pharmacometabolomic profiling of two antipsychotics reveals region-specific neurochemical alterations in the rat brain

**DOI:** 10.1016/j.neurot.2026.e01101

**Published:** 2026-10-22

**Authors:** Yea-Rin Lee, Nivetha Dayanidhi, Irena Loryan, Margareta Hammarlund-Udenaes, Reza Shariatgorji, Anna Nilsson, Per E. Andrén, Theodosia Vallianatou

**Affiliations:** a Department of Pharmaceutical Biosciences, Spatial Mass Spectrometry, Uppsala University, Uppsala, SE-75124, Sweden; b Department of Pharmacy, Translational PKPD Research Group, Uppsala University, Uppsala, SE-75123, Sweden; c Spatial Mass Spectrometry, Science for Life Laboratory, Uppsala University, Uppsala, SE-75124, Sweden; d Wits Integrated Molecular Physiology Research Initiative, Department of Physiology, School of Biomedical Sciences, Faculty of Health Sciences, University of the Witwatersrand, Johannesburg, South Africa

**Keywords:** Pharmacometabolomics, Brain imaging, Mass spectrometry, Antipsychotics

## Abstract

Defining how antipsychotic drugs acutely reshape neurochemical signaling across discrete brain regions is critical for understanding their central pharmacological actions. Matrix-assisted laser desorption/ionization mass spectrometry imaging (MALDI-MSI) enables spatially resolved, untargeted molecular profiling directly in tissue and is well suited to investigate early drug-induced effects. Here, we applied MALDI-MSI to characterize acute neurochemical and metabolomic alterations and to explore trypsin-digested peptidomic changes in rat brain following single-dose exposure to the atypical antipsychotics risperidone and olanzapine. By using MALDI matrices optimized for neurotransmitters, small metabolites, and peptides, we achieved broad molecular coverage across cortical, striatal, and hippocampal regions. This approach revealed distinct, drug-specific spatial signatures encompassing monoamine-related metabolites, purine metabolism, and lipids. In the striatum, both compounds altered dopamine metabolism, as reflected by changes in 3-methoxytyramine, 3,4-dihydroxyphenylacetic acid, and homovanillic acid. In cortical and hippocampal regions, risperidone selectively increased levels of the norepinephrine metabolite 3-methoxy-4-hydroxyphenylethyleneglycol sulfate, indicating a distinct noradrenergic signature. Pathway analysis identified purine metabolism as a major treatment-responsive pathway, consistent with altered nucleotide homeostasis and energy metabolism. Together, these findings provide spatially resolved insight into how atypical antipsychotics modulate brain chemistry, revealing region-specific neurochemical signatures underlying their pharmacological effects.

## Graphical abstract

**Figure:**
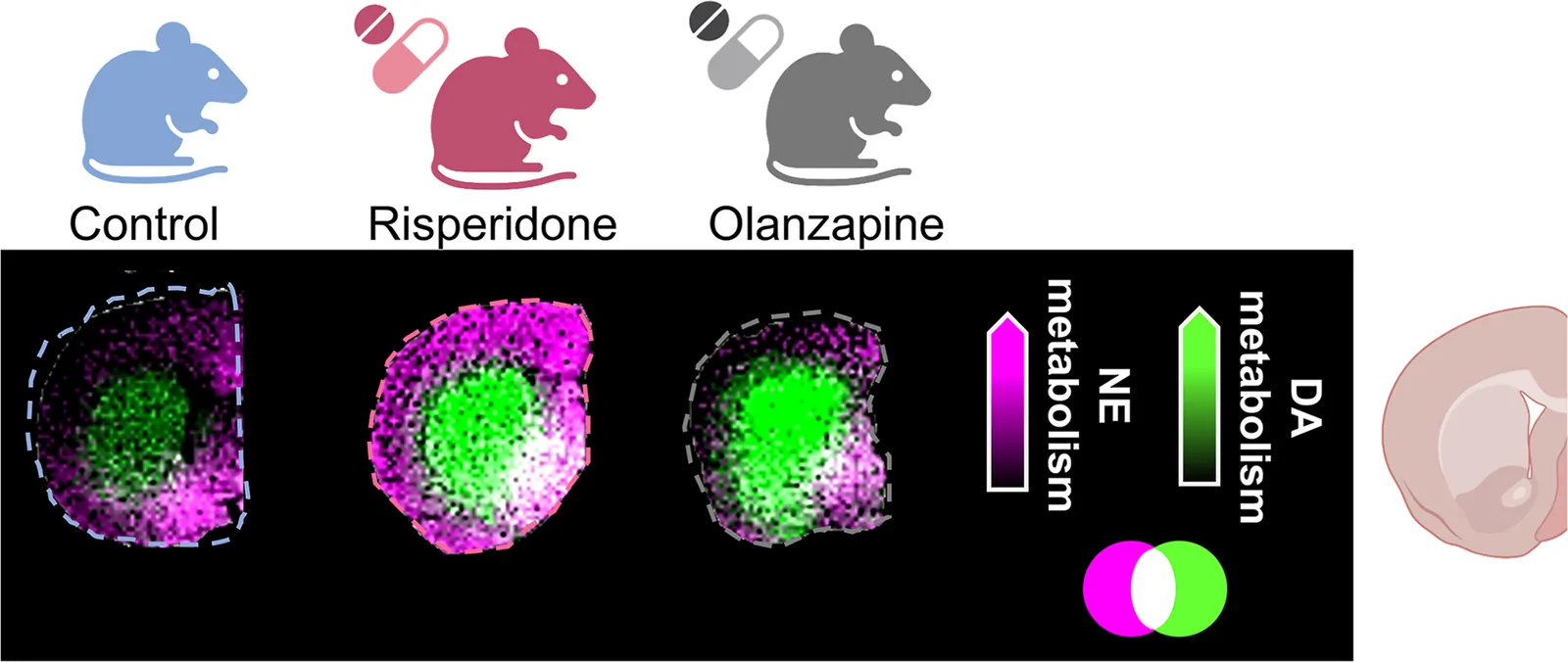

## Introduction

The management of schizophrenia and related psychiatric disorders has been significantly transformed by the development of antipsychotic drugs. Typical antipsychotics are effective in controlling acute positive symptoms such as hallucinations and delusions [1]. However, their use is often constrained by a high risk of extrapyramidal side effects, including tardive dyskinesia, which commonly lead to treatment discontinuation [2], [3], [4], [5], [6], [7]. In contrast, atypical antipsychotics, such as risperidone (RIS) and olanzapine (OLZ), have reshaped clinical practice by offering a lower risk of extrapyramidal symptoms and demonstrating potential cognitive benefits [8], [9], [10], [11]. Despite these advantages, the growing use of atypical antipsychotics has been accompanied by an increase in overdose cases, highlighting the ongoing need to carefully evaluate their benefit-risk profile in clinical settings [12]. Furthermore, the recent approval of xanomeline/trospium—the first neuroleptic drug targeting the cholinergic system—underscores both the complexity and the gaps in our understanding of the neuropathology of schizophrenia [13].

Beyond symptom management, there is growing interest in understanding the broader neurochemical and metabolic consequences of atypical antipsychotic use [14]. These drugs can substantially alter neurotransmitters systems and their associated metabolic pathways in the brain, necessitating careful evaluation of both their short- and long-term pharmacological effects. Previous studies have shown that atypical antipsychotics influence brain structure, cognitive function, neurometabolic activity, and stress-response pathways [15], [16], [17], [18], [19], [20], [21], [22], [23], [24]. However, these studies typically involve patients receiving prolonged treatments with heterogenous drug regimens and variable dosing strategies, complicating the interpretation of drug-specific effects [17], [18], [25], [26]. In particular, there remains a significant gap in understanding the acute neurochemical and metabolic effects of individual atypical antipsychotics, as most studies have prioritized chronic exposure or grouped multiple drugs together without accounting for their distinct pharmacodynamic profiles. Furthermore, existing studies often emphasize clinical efficacy in relation to systemic metabolic side effects, such as dyslipidemia, hyperglycemia, and insulin resistance [27], [28], [29]. These limitations underscore the need for refined experimental approaches to precisely characterize how individual atypical antipsychotics induce neurochemical and metabolic alterations within specific brain regions, particularly during the early phase of treatment.

Matrix-assisted laser desorption/ionization mass spectrometry imaging (MALDI-MSI) has emerged as a powerful tool for investigating spatially resolved biochemical changes induced by drug treatments [8], [30], [31], [32], [33]. By enabling spatial mapping of neurotransmitters, metabolites, and tryptic peptides within specific brain regions, MALDI-MSI provides unique molecular insights into the neurochemical effects of antipsychotic therapies. Building on previous studies of regional blood-brain barrier (BBB) transport of RIS and OLZ, expressed as Kp,uu -the extent of unbound drug transport across the BBB [34,35], the present study utilizes an advanced multi-matrix MALDI-MSI approach to systematically profile region-specific metabolomic and trypsin-digested peptidomic alterations. We focus on the striatal and hippocampal regions, which are critically involved in neurotransmitter signaling and represent key targets of antipsychotic drug action. Utilizing Fourier transform ion cyclotron resonance (FTICR) and/or trapped ion mobility spectrometry time-of-flight (timsTOF) mass spectrometry, we achieve high mass and high spatial resolution imaging to characterize neurochemical signatures associated with RIS and OLZ treatment. This comprehensive analysis aims to enhance our understanding of the molecular pathways underlying efficacy driving mechanisms of atypical antipsychotic action and inform strategies to mitigate their adverse metabolic effects.

## Material and methods

### Chemicals and reagents

OLZ, RIS, 2-hydroxypropyl-β-cyclodextrin (HPβCD), and isopentane were obtained from Sigma-Aldrich (Stockholm, Sweden). Acetonitrile (ACN), acetic acid, methanol (MeOH), trifluoroacetic acid (TFA), along with cresyl violet, were sourced from Merck (Darmstadt, Germany). Absolute ethanol was supplied by VWR (Stockholm, Sweden). Water used in all experiments was either high-performance liquid chromatography (HPLC) grade (Merck, Darmstadt, Germany) or purified *via* a Milli-Q system (Millipore, Bedford, MA, USA). For matrix applications, 2,5-dihydroxybenzoic acid (DHB), 9-aminoacridine (9-AA), and alpha-cyano-4-hydroxycinnamic acid (CHCA) were obtained from both Merck and Sigma-Aldrich. The reactive matrix 4-(anthracen-9-yl)-2-fluoro-1-methylpyridin-1-ium iodide (FMP-10) was acquired from Tag-ON AB (Uppsala, Sweden). Trypsin gold enzyme was purchased from Promega (Madison, WI, USA).

### Animal experiments

Animal experiments were conducted in accordance with protocols established in previous *in-vivo* MALDI-MSI neuropharmacokinetic (neuroPK) studies [34]. Briefly, a total of 15 male Sprague-Dawley rats (250-300 g; Taconic, Lille Skensved, Denmark) were used in the present study. The rats were divided into three groups: six received OLZ (*n =* 6), six were administered RIS (*n* = 6), and three were given the vehicle treated as controls (CTRL; *n* = 3).

Both OLZ and RIS were administered intravenously in a 10% hydroxypropyl-β-cyclodextrin solution in saline, delivered *via* a fast-rate infusion over 5 min followed by a 4-h constant slow-rate infusion at 1 mL kg^−1^ h^−1^ (Uppsala University Hospital Pharmacy, Uppsala, Sweden). Due to the relatively low detection sensitivity of MSI for RIS and OLZ, optimal dosing was adjusted based on previous neuroPK optimization studies [34], [35]. The final total doses were 7.7, and 5 mg kg^−1^ for RIS and OLZ, respectively. CTRL rats received vehicle only, 10% hydroxypropyl beta-cyclodextrin solution in saline, over the same 4-h infusion with identical flow rate. At the end of the infusion, rats were anesthetized with 5% isoflurane (Abbot Scandinavia, Solna, Sweden) for a maximum of 3 min and subsequently decapitated. Brains were promptly removed, sagittally bisected, flash-frozen in isopentane pre-chilled on dry ice for 10 s, wrapped in aluminum foil, and stored on dry ice before being transferred to -80 °C for subsequent cryo-sectioning.

The total drug concentrations at steady state in plasma were previously measured to be approx. 2000 ng/g and 150 ng/g for RIS and OLZ, respectively. The corresponding whole brain total concentrations were 233.4 ± 36.8 ng/g and 724.8 ± 164.3 ng/g, for RIS and OLZ, respectively. For both drugs, the highest regional total concentration was measured in the insulate cortex, 301.9 ± 59.5 ng/g and 1063 ± 239 ng/g, for RIS and OLZ, respectively [34].

The experiments were conducted in compliance with the National Board for Laboratory Animals guidelines and were approved by the Animal Ethics Committee of Uppsala, Sweden (approval number: C189/14). All rats were housed in groups under controlled environmental conditions (20-22 °C, 45-65% humidity) with a 12-hr light/dark cycle and *ad libitum* access to food and water. The animals were allowed to acclimatize for one week prior to the experiments.

### Tissue preparation

Rat brain tissues were sectioned at -20 °C using a cryostat-microtome (CM1900, Leica Microsystems, Wetzlar, Germany). Coronal sections, 12 μm in thickness, were collected at approximate bregma levels of -0.15 mm and -2.40 mm to highlight the striatal and hippocampal regions, respectively [36]. The sections were thaw-mounted onto indium tin oxide (ITO)-coated glass slides (Bruker Daltonics). A total of 15 sections were prepared across the groups (CTRL, *n* = 3; RIS, *n* = 6; OLZ, *n* = 6) and stored at -80 °C prior to MALDI-MSI analysis. For the analysis of neurotransmitters and metabolites, brain tissue sections from both the striatal and hippocampal levels were used, whereas tryptic peptide analysis was conducted only on sections from the striatal level.

### MALDI-MSI analysis

Following storage, brain tissue sections were thawed in a desiccator for 15 min prior to analysis. For neurotransmitter and metabolite imaging, the matrices FMP-10, 9-AA, and DHB were individually applied to consecutive tissue sections using an automated sprayer, with spray settings detailed in Table S1. For visualization of the brain distribution of OLZ and RIS at the hippocampal level (Fig. S1), not previously demonstrated in the NeuroPK study [34], DHB was used as matrix and a mixture of deuterated OLZ and RIS analogs was additionally applied prior to matrix deposition, serving as internal standards (Table S1). The concentration of each internal standard i.e., 30 ng/mL−1 risperidone-*d*_4_, and 40 ng/mL−1 olanzapine-*d*_8_, was selected to approximate the average signal intensity of the corresponding native compound in the relevant brain tissue sections [34].

After matrix application, all slides were scanned to ensure proper image alignment. For investigating the neurochemical changes of the brain metabolome induced by OLZ and RIS, MALDI-MSI experiments were conducted using a MALDI-FTICR instrument (Solarix XR 7T-2Ω, Bruker Daltonics), equipped with a Smartbeam II 2 kHz laser, given its high mass resolution required for metabolomic profiling. MALDI-MSI of OLZ and RIS was conducted on consecutive brain tissue sections using the continuous accumulation of selected ion (CASI) mode, as previously described [34]. Positive-ion mode was employed for FMP-10 and DHB matrices, while 9-AA was analyzed in negative-ion mode. All instruments were externally calibrated using red phosphorus, and matrix-derived lock-mass ions were used as internal calibrants. Detailed instrument parameters are provided in Table S2.

For tryptic peptide imaging, separate adjacent serial tissue sections were delipidated by serial washing with ice-cold methanol, followed by chloroform. Heat-induced antigen retrieval was then performed in 10 mM citric acid (pH 6.0) using a water bath at 95 °C for 45 min. The sections were subsequently digested with trypsin in a humidified chamber at 37 °C for 4 h, after which the CHCA matrix was applied using an HTX M3-Sprayer (HTX Technologies, LLC, Chapel Hill, NC), as previously described [37]. Peptide data were acquired in positive-ion mode using the timsTOF flex MALDI-2 instrument (Bruker Daltonics), equipped with a Smartbeam 3D 10 kHz laser.Prior to data acquisition, height correction and focus adjustments applied. Neither post-ionization nor trapped ion mobility spectrometry (TIMS) was enabled during acquisition. Tissue sections were analyzed in a randomized order to minimize experimental bias.

### Nissl staining

Following MALDI-MSI, the matrix was removed from tissue sections using 100% ethanol to prepare them for Nissl staining and subsequent tissue morphology assessment. Briefly, sections were fixed with two sequential washes in 100% ethanol for 5 min each, air-dried, and rehydrated in water for 1 min. For staining, sections were immersed in a 0.1% cresyl violet solution (0.25% acetic acid in water) for 10 min. Excess stain was removed by washing with water for 5 min, followed by a graded ethanol series (90%, 95%, and 100%) to enhance contrast.

### Data analysis

MSI data from both FTICR and timsTOF flex analyses were visualized and processed using flexImaging (v.5.1, Bruker Daltonics) and SCiLS Lab (v.2024 Pro, Bruker Daltonics). FTICR data were normalized to the root mean square (RMS), while timsTOF flex data were normalized to the total ion count (TIC), unless otherwise specified. Regions of interest within the striatal (cortex; CTX and caudate putamen; CPu) and hippocampal (cortex; CTX and hippocampus; Hip) brain regions were defined according to a stereotaxic atlas [36].

#### Multivariate analysis

CSV files exported from SCiLS Lab (v.2024 Pro, Bruker Daltonics), containing *n* = 2226 (FMP-10), *n* = 1880 (DHB), and *n* = 2625 (9-AA) *m/z* features, were used for multivariate analysis. SIMCA (v.17.0, Sartorius Stedim Biotech, Umeå, Sweden) was employed using a log_10_-transformed intensity (peak area) dataset. Centering and autoscaling to unit variance were applied to standardize variables, ensuring comparability across all features measured on the same scale. Principal component analysis (PCA) was performed to assess data clustering and detect outliers using Hotelling's T^2^ ellipse at a 95% confidence interval (Fig. S2). Partial least squares discriminant analysis (PLS-DA) was also performed to identify metabolites contributing to group separation. Variable importance of projection (VIP) scores (VIP >1) was then calculated to refine *m/z* lists for further analysis. Model fit and predictability were assessed using R^2^ and Q^2^, respectively, together with the R^2^−Q^2^ difference, CV-ANOVA, and permutation testing. In detail, the derived models were first evaluated by their R^2^ and Q^2^ statistics and, particularly, their difference (R^2^ - Q^2^) which should be < 0.2 units. Subsequently, CV-ANOVA (analysis of variance testing of cross-validated predictive residuals), a diagnostic tool for assessing the reliability of PLS models was implemented. The derived p-value indicates the probability level where a model with this F-value may be the result of just chance (a p-value lower than 0.05 points to a significant model). In addition, permutation tests (100 permutations) were performed for each PLS-DA model, where the corresponding plot displays the correlation coefficient between the original y-variable and the permuted y-variable on the x-axis versus the cumulative R^2^ and Q^2^ on the Y-axis, and draws the regression line. The intercept is a measure of the overfit. All the above criteria were fulfilled by the PLS-DA models used for detecting treatment-induced neurochemical changes (Table S3, Figs. S3–S5).

#### Univariate analysis

In parallel with the multivariate analysis performed on whole-brain tissue level (striatal and hippocampal), untargeted univariate analysis was performed for each investigated brain region (i.e., CTX-striatal level, CTX-hippocampal level, CPu and Hip) using the MetaboAnalyst 5.0 platform (https://www.metaboanalyst.ca). In this case, CSV files were exported from SCiLS Lab per region, containing the same number of *m/z* features as in the multivariate analysis (*n* = 2226 for FMP-10, *n* = 1880 for DHB, and *n* = 2625 for 9-AA). Since FMP-10 is a reactive matrix/derivatization reagent selectively reacting with specific functional groups, it does not provide equal coverage of the whole metabolome and it can generate unidentifiable cluster fragment peaks or underivatized ionizable molecules (e.g., lipids) [38]. Therefore, for FMP-10 derived data, an additional list of 60 structurally validated metabolites was extracted for further analysis [39], [40], [41]. Before analysis, all data were log_10_-transformed for any lack of normality. One-way ANOVA was applied, and the resulting p-values were adjusted for multiple testing using the Benjamini–Hochberg false discovery rate (FDR) procedure. Post-hoc analysis was performed with Fisher's LSD. Commonly significantly features between the multivariate and univariate analyses were selected for structural validation and biological interpretation. For data demonstration, ordinary one-way ANOVA tests, followed by Tukey's multiple-comparisons tests, were applied after verifying normality (Shapiro-Wilk test) and homogeneity (Levene's test). Box plots were generated in GraphPad Prism (v.11, GraphPad Software Inc, San Diego, California, USA), with *p*-values indicated as follows: ∗*p* ≤ 0.05, ∗∗*p* ≤ 0.01, ∗∗∗*p* ≤ 0.001, ∗∗∗∗*p* ≤ 0. 0001. Univariate analysis was also conducted in SCiLS Lab on non log_10_-transformed intensities confirming the findings.

#### z-score heat map analysis

Log_10_-transformed ion intensities from FMP-10, DHB, 9-AA, and tryptic-digested peptide datasets in SCiLS Lab were converted to *z*-scores for comparative visualization across groups. Z-scores were calculated as (abundance - mean abundance)/standard deviation.

#### Volcano plot analysis

Volcano plots were used for untargeted pairwise comparisons between groups (CTRL vs. RIS, CTRL vs. OLZ, and RIS vs. OLZ). Log_2_ fold changes and -log_10_ *p*-values (*t*-test) of normalized peptide intensities were plotted, with significance thresholds set at *p* < 0.05 (-log_10_ *p* > 1.3010) and fold changes >2 (log_2_ fold change > ±1). Significantly altered peptides between groups were highlighted.

#### Protein-protein interaction (PPI) networks and Gene Ontology (GO) pathway analysis

A high-confidence (>0.7) PPI network for differentially expressed genes (DEGs) was constructed using the STRING database (https://string-db.org/), with species set to *Rattus norvegicus*. Network edges represented functional and physical associations, with line thickness indicating the strength of supporting data, derived from text mining, experimental data, curated databases, and co-expression. Proteins were clustered using DBSCAN based on local node density. Additionally, gene Ontology (GO) pathway analysis was also conducted *via* STRING, categorizing significant proteins into three functional classifications such as 1. biological processes (assessing cellular activity changes), 2. molecular functions (evaluating binding and enzymatic interactions), and 3. cellular components (determining subcellular localization). This analysis provided insights into pathways and functions affected by antipsychotic treatments, linking metabolic changes to neuronal structure, function, and energy homeostasis.

### Metabolite identification and pathway analysis

Significantly altered neurotransmitter and metabolites were first identified using The Human Metabolome Database (HMDB; https://hmdb.ca/) and Met-ID (an *in-house* developed open-source software) [42]. Adducts considered for positive-ion mode included [M + H]^+^, [M + Na]^+^, [M + K]^+^, [M + 2Na - H]^+^, [M + 2K - H]^+^, and [M - H_2_O + H]^+^, while negative-ion mode included [M - H]^-^, [M + Cl]^-^, [M + K - 2H]^-^, [M + Na - 2H]^-^, and [M - H_2_O - H]^-^, with a mass error margin of ±4 ppm.

For higher confidence structural validation, on-tissue MS/MS was performed when sufficient precursor ion intensity was available. Precursor ions were isolated using a 1 Da window, followed by quadrupole selection and fragmentation in the collision cell. Collision energies, optimized for each analyte, ranged from 5.0 to 20 V. Product ion spectrum collected on tissue were compared to MS/MS libraries. When MS/MS based structural validation was not feasible due to precursor ion's low abundance, high mass resolution and cross-validation among different detected adducts deriving from the same molecular species were applied. Pixel-by-pixel spatial correlation between different ions coming from the same molecules and evaluation of their statistics used for increasing the identification confidence. The identification of FMP-10 derivatized species was based on our previous extensive work [39], [40], [41]. Additionally, distinct metabolites identified through these analyses were subjected to metabolic pathway analysis using the MetaboAnalyst 5.0 platform (https://www.metaboanalyst.ca).

For tryptic peptide analysis, a different approach was necessary due to the limited availability of MALDI-MSI databases for peptide mass matching. To address this challenge, the SCiLS Lab T-ReX 2 algorithm was used to generate a feature list of 450 singly charged *m/z* values, accurately detecting and aligning the monoisotopic peaks of putative tryptic peptides. These *m/z* values were subsequently matched to their singly charged equivalents derived from doubly charged ions measured in LC-MS/MS data. Calculated *m/z* values were cross verified against theoretical *m/z* values for known peptides obtained from published studies (Fig. S6) [24], [43], [44]. Only protonated [M + H]^+^ analytes with a mass error margin within ±10 ppm were considered for further analysis. The final list of matched peptide masses was then searched against the UniProt database, restricted to *Rattus norvegicus* (taxonomy ID 10116), and filtered to include only reviewed entries from UniProt (Swiss-Prot). Peptide *m/z* values and their maximum intensity data were exported from SCiLS Lab and used to generate volcano plots, *z*-score heat maps, protein-protein interaction (PPI) networks, and Gene Ontology (GO) pathway enrichment analyses (https://string-db.org/). A detailed description of data analyses is provided in the Supporting Information.

Direct on-tissue peptide MS/MS has historically been constrained by limited sensitivity and fragmentation efficiency, as well as interference from matrix-derived ions [45]. Given these limitations, MALDI-MSI peptide features were putatively annotated by accurate-mass matching against an in-house LC–MS/MS reference library, and features exhibiting the smallest mass errors relative to the corresponding reference peptide m/z values were selected for further analysis. The reference library was generated using data-dependent acquisition with parallel accumulation–serial fragmentation (DDA-PASEF) on a timsTOF instrument, as previously described [37].

### Calculation of metabolic ratios as apparent catabolic indices

In order to provide an estimation of the impact of the investigated antipsychotics on certain catabolic processes, given the limitations of a single-dose, steady-state study, we calculated the [product]/[precursor] MSI intensity ratios to visualize spatial differences in their relative abundances for the dopamine and purine pathways. The homovanillic acid (HVA)/dopamine MSI intensity ratio was calculated as an apparent dopamine catabolic index, reflecting the relative balance between dopamine and its downstream metabolite. The DOPAC/3-MT MSI intensity ratio was calculated as an apparent intracellular/extracellular dopamine metabolism index, reflecting the relative balance between dopamine catabolites primarily associated with intracellular (DOPAC) versus extracellular (3-MT) metabolism. The adenosine/ATP MSI intensity ratio was calculated as an apparent ATP breakdown index, reflecting the relative balance between ATP and its downstream catabolite adenosine.

## Results

### Multi-matrix approach enhances neurochemical detection

Different MALDI matrices offer distinct advantages depending on the physicochemical properties of the target metabolites and neurotransmitters, as ionization efficiency varies between positive- and negative-ion modes. In this study, both 9-AA and DHB matrices were employed to facilitate the detection of small-molecule metabolites (Fig. 1a). The 9-AA matrix demonstrated superior performance in ionizing low-molecular-weight compounds in negative-ion mode, while DHB enabled broad-spectrum metabolite detection in positive-ion mode [46]. Notably, DHB also proved effective in detecting neuroleptic compounds, including RIS and OLZ, as well as the active metabolite 9-hydroxyrisperidone, i.e., paliperidone, in the brain tissue of RIS-treated animals (Fig. 1b) [47]. While OLZ was widely distributed in the grey matter regions of the rat brain tissue, RIS and its metabolite demonstrated higher localization in the lateral and third ventricles of the brain.

**Fig. 1:**
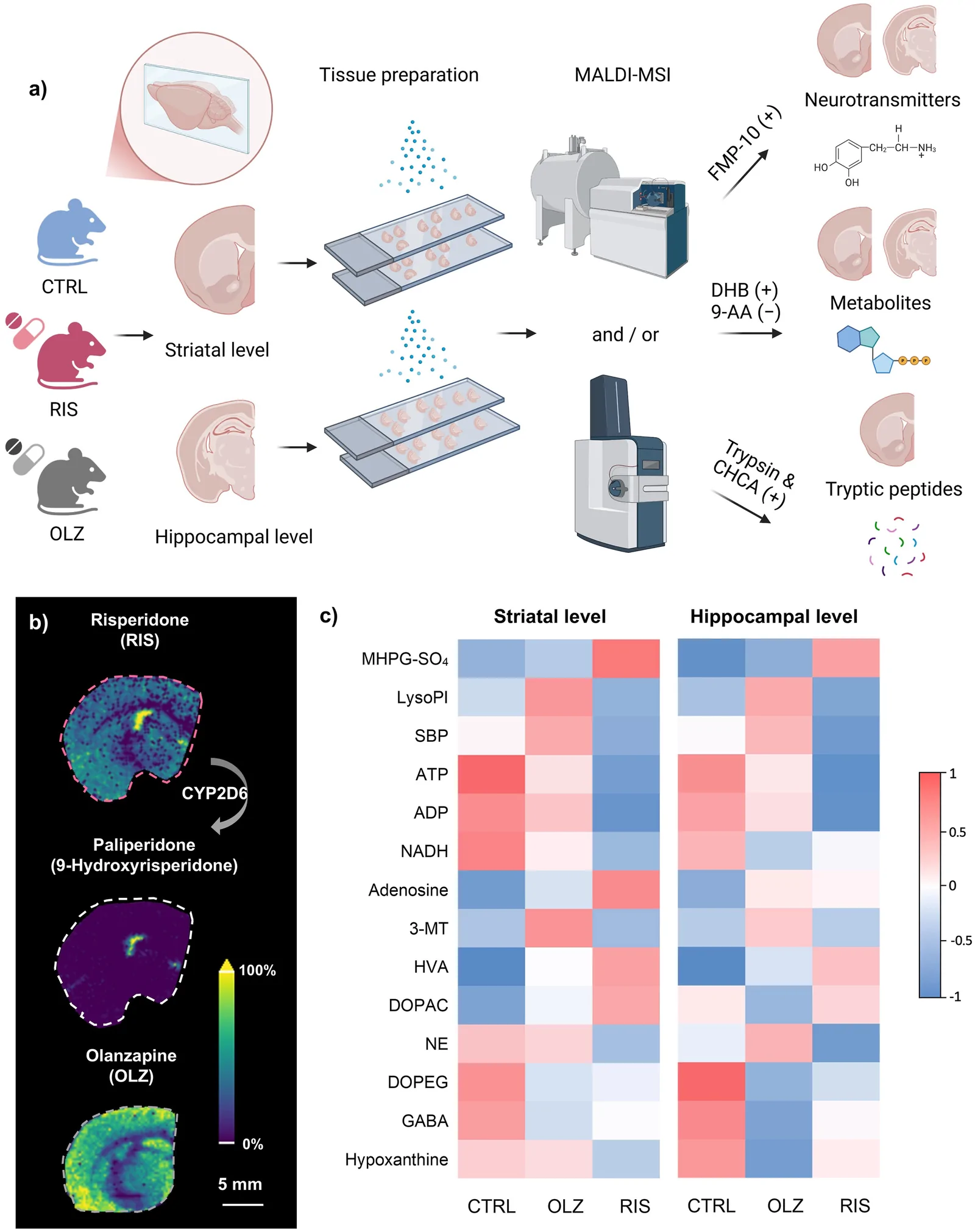
**Multi-matrix MALDI-MSI approach for the detection of multiple neurochemical species** (a) Overview of the MALDI-MSI experimental workflow. Tissue sections from each group, i.e., non-treated controls (CTRL; *n* = 3), risperidone- (RIS; 7.7 mg kg^−1^; *n* = 6) and olanzapine-treated (OLZ; 5 mg kg^−1^; *n* = 6), were mounted onto two ITO-coated slides corresponding to the striatal and hippocampal brain regions. FMP-10, DHB, and 9-AA matrices were applied for neurotransmitter and metabolite detection, enabling spatial distribution analysis. For tryptic peptide imaging, tissues underwent delipidation and heat-induced antigen-retrieval, followed by enzymatic digestion with trypsin and CHCA matrix application. (b) Representative MALDI-MSI ion images illustrating the spatial distribution of risperidone (RIS; 7.7 mg kg^−1^, *m/z* 411.218) and paliperidone (9-Hydroxyrisperidone, *m/z* 427.213), both normalized to risperidone-*d*_4_ (peak maximum of *m/z* 415.244 ± 0.00076 Da), and olanzapine (OLZ; 5 mg kg^−1^, *m/z* 313.148), normalized to olanzapine-*d*_8_ (peak maximum of *m/z* 321.198 ± 0.00052 Da). Additional ion images are provided in Fig. S1. CYP2D6 stands for the cytochrome P450 2D6 enzyme, the major enzyme involved in the conversion of risperidone to paliperidone [47]. The whole brain total concentrations were 233.4 ± 36.8 ng/g and 724.8 ± 164.3 ng/g, for RIS and OLZ, respectively, as measured in the previous *in-vivo* neuropharmacokinetic (neuroPK) study [34]. (c) Heat maps generated using *z*-scores for statistically significant *m/z* features corresponding to neurotransmitters and metabolites, comparing groups at the striatal and hippocampal levels. A complete list of *m/z* features and their corresponding metabolite identities is in Table S4.

For neurotransmitter analysis, FMP-10 was used due to its selective reactivity with phenolic moieties and primary amines, allowing for highly specific detection of catecholaminergic neurotransmitters in positive-ion mode [33].

PCA was first performed on each brain level to assess group clustering and detect potential outliers (Fig. S2). PLS-DA was subsequently applied to identify metabolites contributing to group separation. Based on the multivariate criterion of VIP >1, lists of discriminant *m/z* features were separately generated for the 9-AA, DHB, and FMP-10 datasets. These features were then subjected to univariate analysis (one-way ANOVA followed by Tukey's multiple tests, *p* < 0.05) to identify statistically significant ions in specific brain regions, i.e., CTX, CPu, and Hip. Individual *m/z* features passing both multivariate and univariate thresholds were further investigated for putative metabolite identification and verification of spatial distribution *via* ion images, resulting in finalized metabolite lists. In total, 14 metabolites displayed significant differences across CTRL, RIS, and OLZ groups, encompassing neurotransmitter, nucleotide, and lipid classes (Fig. 1c). The FDR values of these metabolites in specific brain regions, derived by the untargeted one-way ANOVA, are provided in Table S5 (*FDR< 0.05, unless otherwise stated*).

Seven neurotransmitters and their related metabolites—homovanillic acid (HVA), 3,4-dihydroxyphenylacetic acid (DOPAC), 3-methoxy-4-hydroxyphenylethyleneglycol sulfate (MHPG-SO_4_), γ-aminobutyric acid (GABA), 3-methoxytyramine (3-MT), norepinephrine (NE) and 3,4-dihydroxyphenylethylene glycol (DOPEG)—revealed alterations in catecholaminergic and GABAergic neurotransmission (Fig. 1c). Five nucleotide and energy-related metabolites—adenosine, hypoxanthine, adenosine diphosphate (ADP), adenosine triphosphate (ATP), and nicotinamide adenine dinucleotide (NADH)—suggested that antipsychotic treatment disturbs nucleotide metabolism and mitochondrial redox homeostasis (Fig. 1c). Additionally, two metabolites associated with oxidative stress and lipid regulation—sedoheptulose 1,7-bisphosphate (SBP), and lysophosphatidylinositol (LysoPI (20:4))—indicated treatment-dependent changes in oxidative stress responses, pentose phosphate pathway activity, and membrane lipid remodeling (Fig. 1c).

### Alterations in neurotransmitter metabolism

Although striatal dopamine levels were not significantly altered by either neuroactive agent, dopamine metabolites, i.e., 3-MT, DOPAC, and HVA, were significantly elevated by both OLZ and RIS in distinct patterns, as demonstrated by one-way ANOVA followed by Tukey's multiple-comparisons tests (Fig. 2a,c). Notably, 3-MT, which is formed *via* catechol-*O*-methyl transferase (COMT)-mediated methylation of dopamine in the extraneuronal space, was significantly elevated in the CPu of OLZ-treated brains compared to CTRL. In contrast, the striatal levels of DOPAC, the oxidized metabolite of dopamine synthesized *via* monoamine oxidase A/B (MAO-A/B) intraneuronally, were more intensely elevated by RIS [34] (FDR= 0.156, Table S5). The striatal ratio of DOPAC/3-MT, an indicator of apparent intracellular/extracellular dopamine metabolism, was significantly elevated by RIS. However, the overall apparent metabolism of dopamine in the CPu, expressed as HVA/DA, was significantly elevated by both drugs (Fig. 2a,c).

**Fig. 2:**
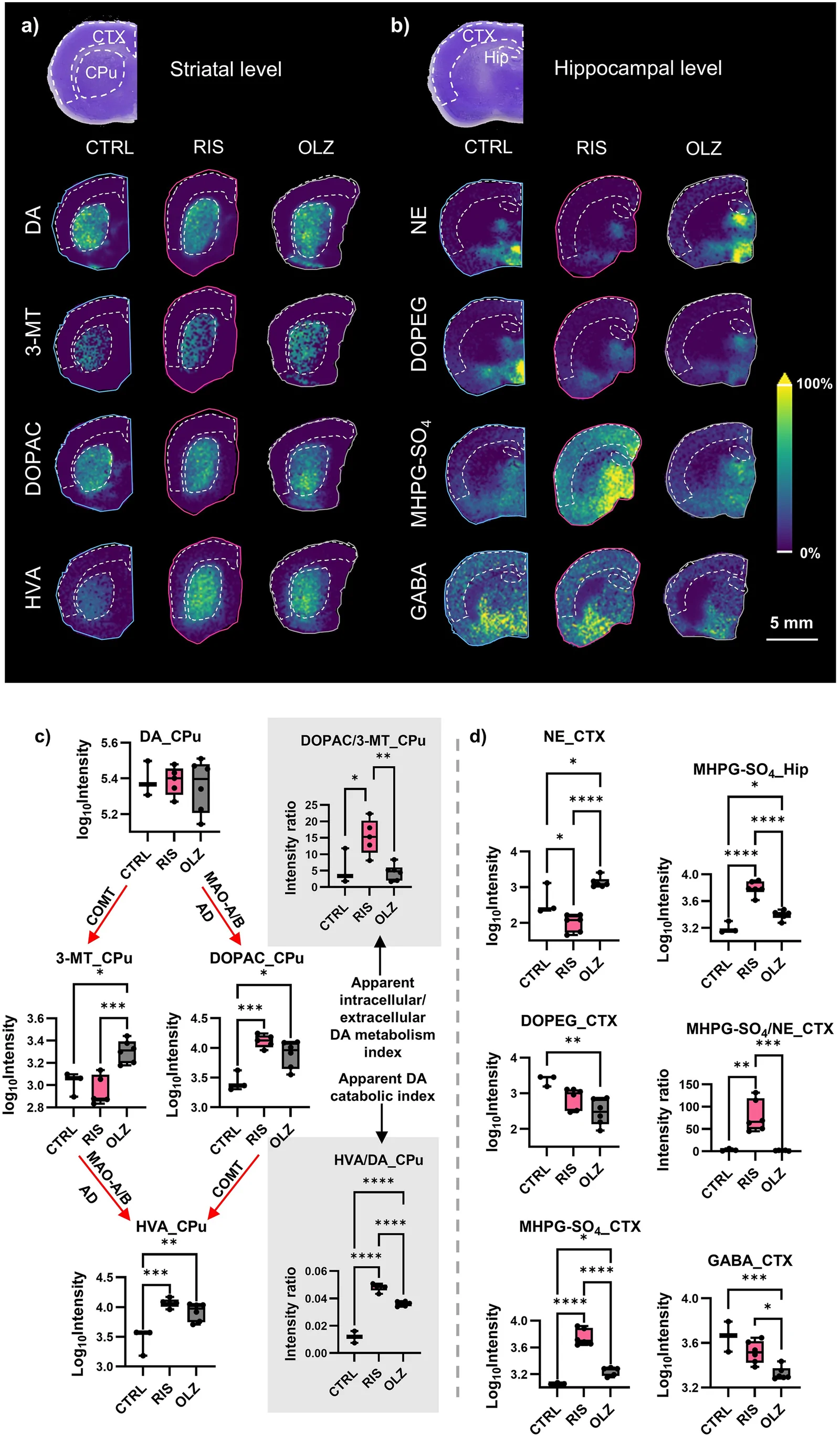
**Region-specific alterations in neurotransmitters and related metabolism following RIS (7.7 mg kg^−1^) and OLZ (5 mg kg^−1^) treatment** (a) Representative MALDI-MSI ion images of DA (*m/z* 674.280, FMP-10), 3-MT (*m/z* 435.207, FMP-10), DOPAC (*m/z* 689.244, FMP-10), and HVA (*m/z* 450.170, FMP-10) in striatal level regions (CTX and CPu) across CTRL, RIS, and OLZ groups. (b) Representative MALDI-MSI ion images of NE (*m/z* 690.275, FMP-10), DOPEG (*m/z* 691.259, FMP-10), MHPG-SO_4_ (*m/z* 263.023, 9-AA), and GABA (*m/z* 371.175, FMP-10) in hippocampal level regions (CTX and Hip) across CTRL, RIS, and OLZ groups. (c) Representative box plots showing log_10_-normalized intensity of dopaminergic metabolites in the CPu. (d) Representative box plots showing log_10_-normalized intensity of noradrenergic and GABEergic metabolites in CTX and Hip. The ratio MHPG-SO_4_ expresses the overall noradrenergic metabolism. Box plots indicate statistically significant group differences after one-way ANOVA followed by Tukey's correction for multiple comparisons, presented by asterisks: ∗*p* ≤ 0.05, and ∗∗*p* ≤ 0.01, ∗∗∗*p* ≤ 0.001, ∗∗∗∗*p* ≤ 0.0001. Ion images were normalized using root mean square (RMS) and acquired at 100 μm spatial resolution. Abbreviations: dopamine (DA), 3-methoxytyramine (3-MT), 3,4-dihydroxyphenylacetic acid (DOPAC), homovanillic acid (HVA), 3-methoxy-4-hydroxyphenylglycol sulfate (MHPG-SO_4_), norepinephrine (NE), 3,4-dihydroxyphenylethylene glycol (DOPEG), and gamma-aminobutyric acid (GABA), catechol-*O*-methyl transferase (COMT), monoaminoxidase A/B (MAO-A/B).

Noradrenergic metabolic alterations were observed after RIS administration in CTX and Hip, expressed as significantly increased levels of MHPG-SO_4_ (Fig. 2a,b,d). This observation led to thorough structural validation of MHPG-SO_4_, as not previously reported with MALDI-MSI, as well as investigation of other sulfated catecholaminergic metabolites (Fig. S7). Besides MHPG-SO_4_, DOPEG-SO_4_ and DOPAC-SO_4_ were significantly affected in the CTX and CPu, respectively, the former only by RIS administration whereas the latter by both drugs (Figs. S7 and S8).

Inhibitory neurotransmission was also affected. GABA levels significantly decreased in the CTX following OLZ treatment compared to CTRL, whereas no significant changes were observed in the Hip regions at the hippocampal level (Fig. 2b,d). In addition to these alterations in inhibitory systems, serotonergic signaling may also be affected [48]. A non-significant trend toward reduced serotonin (5-HT) metabolism was also observed in the hippocampal CTX and Hip of OLZ-treated brains compared to CTRL and RIS, suggesting subtle modulation of serotonergic activity (Fig. S9).

### Alterations in nucleotide, and energy metabolism

Adenosine brain metabolism was highly affected by RIS administration (Fig. 3a) and, to a lower extent, by OLZ. RIS-treated brains exhibited a significant reduction in ADP levels in the CTX and CPu, relative to CTRL and OLZ (Fig. 3a,c). A less pronounced reduction was observed in hippocampal regions (Fig. 3b,d). RIS treatment also resulted in a significant reduction in ATP across cortical, striatal and hippocampal regions (Fig. 3a–d). RIS appeared to induce a more pronounced suppression of nucleotide breakdown relative to OLZ. Hypoxanthine levels were significantly reduced in the CTX and Hip at the hippocampus level in OLZ-treated brains compared to RIS, suggesting alterations in ATP catabolism and disruptions in cellular energy metabolism (Fig. 3b,d). Adenosine and ADP were structurally validated by tandem MS (Figs. S10 and S11), while identification of ATP was based on multiple-adduct high spatial correlation (Fig. S12). The adenine containing coenzyme NADH levels were significantly reduced in the CPu of RIS-treated brains compared to CTRL and OLZ (Fig. 3a,c) (FDR= 0.077, Table S5). Additionally, a significant decrease in the striatal levels of *S*-adenosyl methionine (SAM), an adenosine-containing co-substrate involved in transmethylations, was induced by both drugs (Fig. S13). By applying pathway enrichment analysis on all identified metabolites, purine metabolism was highlighted as the most significantly altered metabolic pathway by antipsychotic administration (Fig. S14).

**Fig. 3:**
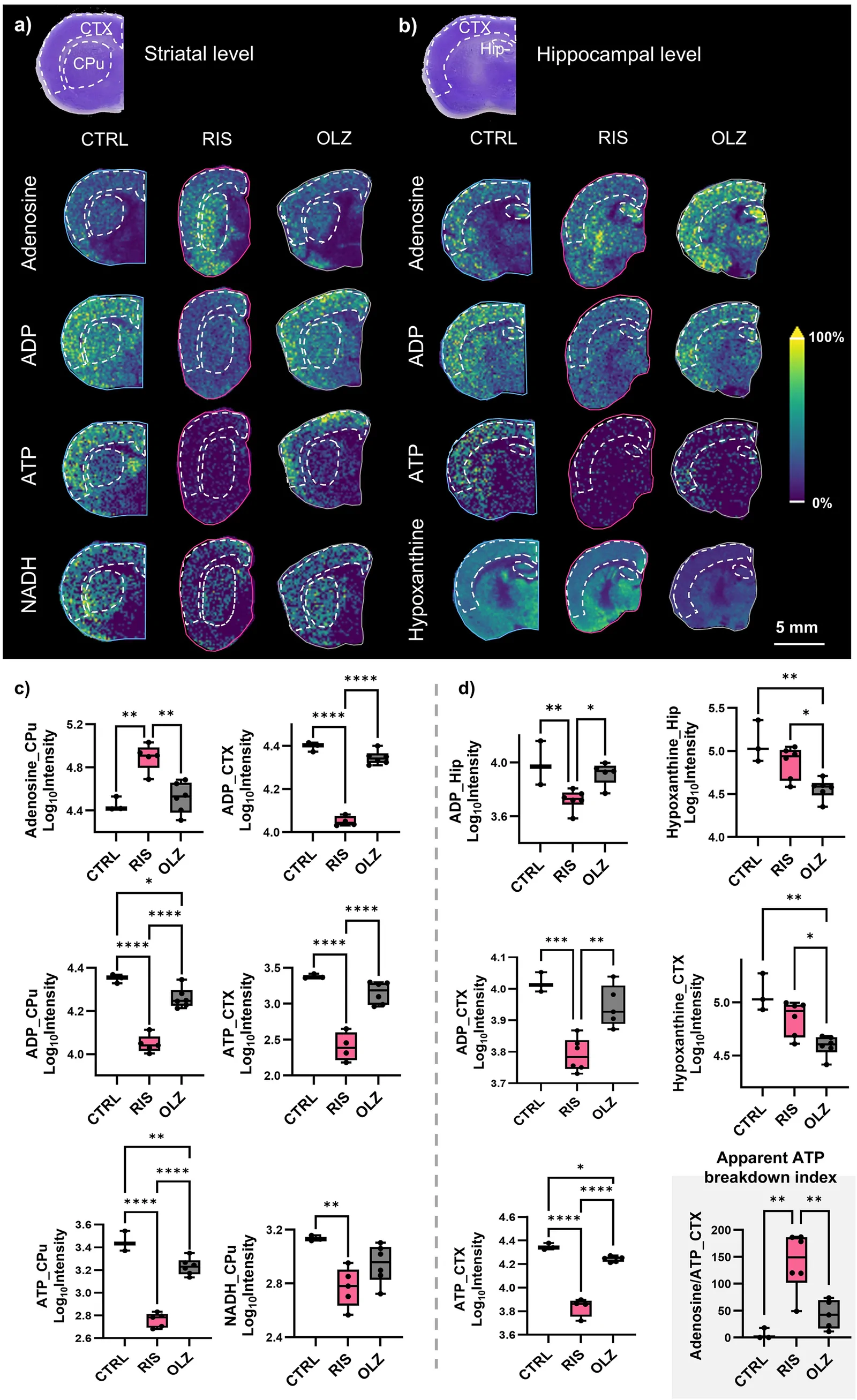
**Region-specific alterations in nucleotide, and energy and oxidative-related metabolism following RIS (7.7 mg kg^−1^) and OLZ (5 mg kg^−1^) treatment** (a) Representative MALDI-MSI ion images of adenosine (*m/z* 268.104, DHB), ADP (*m/z* 465.993, DHB), ATP (*m/z* 583.915, DHB), and NADH (*m/z* 742.044, DHB) in striatal level regions (CTX and CPu) across CTRL, RIS, and OLZ groups. (b) Representative MALDI-MSI ion images of adenosine (*m/z* 268.104, DHB), ADP (*m/z* 465.993, DHB), ATP (*m/z* 545.959, DHB), and hypoxanthine (*m/z* 404.151, FMP-10) in hippocampal level regions (CTX and Hip) across CTRL, RIS, and OLZ groups. (c) Representative box plots showing log_10_-normalized intensity of adenosine, ADP, ATP, and NADH in the striatal level CTX and CPu. (d) Representative box plots showing log_10_-normalized intensity of ADP, ATP, hypoxanthine, and the apparent ATP breakdown index (ratio adenosine/ATP) in the hippocampal level CTX and Hip. Box plots indicate statistically significant group differences, after one-way ANOVA followed by Tukey's correction for multiple comparisons, presented by asterisks: ∗*p* ≤ 0.05, and ∗∗*p* ≤ 0.01, ∗∗∗*p* ≤ 0.001, ∗∗∗∗*p* ≤ 0.0001. Ion images were normalized using RMS and acquired at 100 μm spatial resolution. Abbreviations: adenosine diphosphate (ADP), adenosine triphosphate (ATP), nicotinamide adenine dinucleotide (NADH), and adenosine triphosphate (ATP).

### Alterations in oxidative stress and lipid metabolism

SBP, a key intermediate in the pentose phosphate pathway (PPP) responsible for generating nicotinamide adenine dinucleotide phosphate (NADPH), was significantly reduced in RIS-treated brains compared to OLZ in both the hippocampal CTX and Hip (Fig. 4). Owing to the difficulty in isolating and fragmenting SPB, structural validation on this metabolite was based on spatial correlation and consistent statistics among multiple SBP-related molecular species (Figs. S15 and S16).

**Fig. 4:**
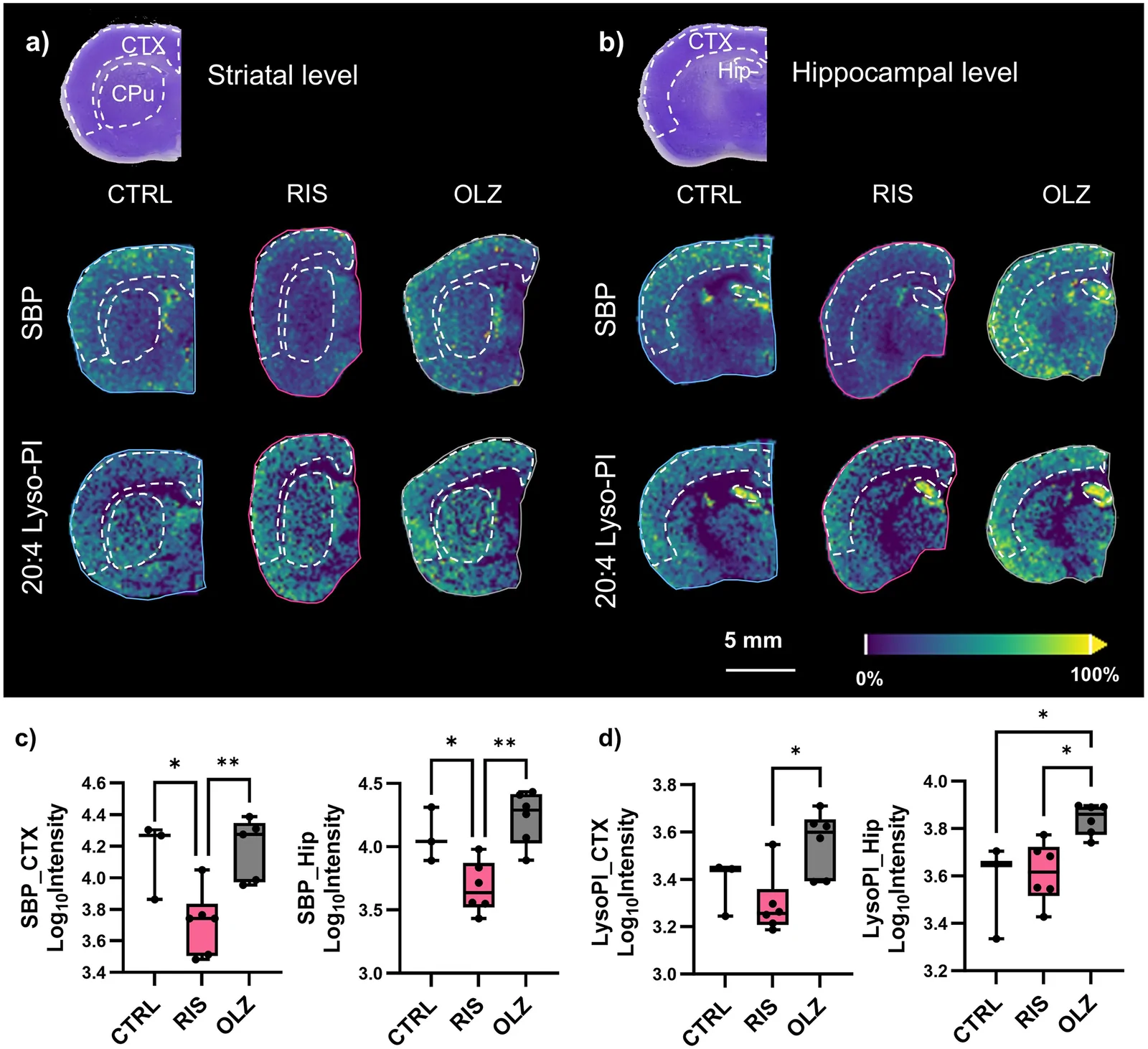
**Region-specific alterations in oxidative stress and lipid metabolism following RIS (7.7 mg kg^−1^) and OLZ (5 mg kg^−1^) treatment** (a) Representative MALDI-MSI ion images of SBP (*m/z* 368.999, 9AA), and LysoPI (20:4) (*m/z* 619.288, 9AA) in striatal regions (CTX and CPu) across CTRL, RIS, and OLZ groups. (b) Representative MALDI-MSI ion images of SBP (*m/z* 368.999, 9AA), and LysoPI (20:4) (*m/z* 619.288, 9AA) in hippocampal regions (CTX and Hip) across CTRL, RIS, and OLZ groups. (c) Representative box plots showing log_10_-normalized intensity of SBP, and LysoPI (20:4) in the striatal CTX and CPu. (d) Representative box plots showing log_10_-normalized intensity of SBP, and LysoPI (20:4) in the hippocampal CTX and Hip. Box plots indicate statistically significant group differences, after one-way ANOVA followed by Tukey's correction for multiple comparisons, presented by asterisks: ∗*p* ≤ 0.05, and ∗∗*p* ≤ 0.01, ∗∗∗*p* ≤ 0.001, ∗∗∗∗*p* ≤ 0.0001. Ion images were normalized using RMS and acquired at 100 μm spatial resolution. Abbreviations: Sedoheptulose 1,7-bisphosphate (SBP), and lysophosphatidylinositol (LysoPI (20:4)).

Metabolomic profiling further revealed significant alterations in lipid metabolism, particularly in LysoPI (20:4), which was identified based on accurate mass. OLZ-treated brains exhibited significantly higher levels of LysoPI (20:4) compared to RIS in the striatal CTX and hippocampal Hip, suggesting increased phospholipid metabolism or enhanced phospholipase activity (Fig. 4).

### PPI networks and GO pathway analyses reveal shared molecular effects

Following tryptic peptide MALDI-MSI analysis, m/z features were extracted using SCiLS Lab and cross-referenced with an in-house LC-MS/MS reference list generated by searching against reviewed Rattus norvegicus entries in the UniProtKB/Swiss-Prot database. This produced a refined list of putatively assigned peptides and proteins (Table S6). Volcano plots and z-score heat maps indicated regional abundance trends, although no individual peptide or protein reached statistical significance (Table S7; Figs. S17 and S18). This may reflect the limited sample size and the preferential detection of higher-abundance tryptic peptides by MALDI-MSI. Nevertheless, the RIS- and OLZ-treated groups showed overlapping peptide and protein profiles, with similar trends relative to the CTRL group.

Exploratory PPI and GO analyses of the shared proteins identified six PPI clusters broadly associated with cytoskeletal organization, synaptic endocytosis, mitochondrial function, and taurine metabolism, together with enriched GO categories related to biological regulation, intracellular organelles, and protein binding (Fig. S19). These patterns were consistent with the previously observed metabolic changes involving energy metabolism, oxidative stress, and neurotransmitter regulation. Proteins associated with BAD activation, mitochondrial translocation, and synaptic endocytosis also suggested possible links to changes in ATP, ADP, and GTP levels and to synaptic plasticity [49], [50], [51]. These findings highlight potential shared molecular processes for further investigation.

## Discussion

In this study, we employed MALDI-MSI using several target-specific matrices to investigate the short-term neurochemical effects of two atypical antipsychotics, i.e., RIS (7.7 mg kg^−1^) and OLZ (5 mg kg^−1^), in the rat brain. These drugs differ in receptor binding profiles and clinical side-effect patterns. Collectively, our findings demonstrate that both drugs induce region-specific metabolic alterations following short-term exposure, with RIS and OLZ producing distinct molecular signatures, particularly in pathways related to neurotransmitter metabolism, cellular energy homeostasis, and oxidative stress regulation. These highlight the capacity of MALDI-MSI to resolve early pharmacologically driven biochemical alterations with high spatial resolution and provide valuable insights into the immediate neurochemical mechanisms underlying atypical antipsychotic pharmacological activity, including efficacy and adverse effects.

Elevation of the overall striatal metabolism of dopamine, expressed as the ratio HVA/DA, was detected after administration of RIS and OLZ, without significant changes in dopamine levels. However, the two drugs demonstrated distinct profiles of apparent intracellular/extracellular dopamine metabolism index DOPAC/3-MT. OLZ preferentially elevated 3-MT, a COMT-mediated extracellular dopamine metabolite, suggesting increased extracellular dopamine availability. In contrast, RIS favored intra-neuronal degradation *via* MAO-A/B, as indicated by the selectively elevated DOPAC/3-MT ratio in the striatum, likely reflecting enhanced dopamine reuptake and intracellular metabolism. Overall, RIS demonstrated a more profound effect on dopamine metabolism. This can be linked to dopamine D_2_ receptor occupancy, since antagonism of presynaptic D2 autoreceptors disrupts inhibitory feedback on dopaminergic neurons, resulting in increased firing and dopamine metabolism [52]. As previously shown, D_2_ receptor occupancy in the striatum was higher for RIS compared to OLZ (K_i,striatum_ 3.3 nM vs 17 nM, respectively) [35], [53]. Since receptor occupancy is driven by both pharmacokinetic and pharmacodynamic characteristics, it should be noted that, for the same animals, higher total CPu concentrations were measured for OLZ compared to RIS (740.8 ± 185.9 vs 267.5 ± 47.3, respectively) as well as *K*_p,uu_ in the same brain region (0.87 ± 0.22 vs 0.10 ± 0.02, respectively) [34].Other studies also support this finding, with RIS reported to exhibit slightly higher D_2_ receptor occupancy than OLZ [54], [55]. Interestingly, RIS has also demonstrated dose-dependent effects on dopamine dynamics within clinically relevant dosing ranges, further reinforcing its modulatory influence on striatal dopaminergic activity [55], [56]. Similarly, the increase in MHPG-SO_4_ and decrease in norepinephrine (NE) supports enhanced NE catabolism, potentially *via* α_2_-adrenergic antagonism, which removes presynaptic inhibition and facilitates NE release followed by rapid metabolism [57]. These findings align with known profiles of RIS and similar D_2_/α_2_-active antipsychotics and reflect higher monoaminergic tone and metabolic activity in dopaminergic and noradrenergic systems. Consistent with this, prior studies have shown that RIS increases NE and its metabolites, indicating heightened noradrenergic activity [58]. It should also be noted that the measured cortical total concentrations of RIS, as well as its *K*_p,uu_ in the cortex were significantly lower compared to OLZ [34].

In contrast, OLZ reduced brain levels of GABA, consistent with previous reports that chronic OLZ administration reduces GABA receptor density in the CTX and Hip, potentially disrupting inhibitory synaptic transmission [59], [60].This profile suggests that OLZ engages inhibitory neuromodulation, reducing global cortical excitability without significantly activating energy-demanding monoaminergic circuits.

Both drugs disrupted striatal ATP and ADP levels, with RIS producing a more marked reduction in ATP and a reciprocal rise in adenosine. This shift in the adenosine/ATP ratio, an index of apparent ATP breakdown, may indicate enhanced adenine nucleotide turnover, possibly due to upregulated activity of purine-catabolizing enzymes such as nucleotidases and nucleoside phosphorylases [61].This may reflect high neural energy demand due to increased firing and neurotransmitter recycling. Adenosine accumulation under metabolic stress is a well-known phenomenon that activates A_1_ and A_2A_ receptors, modulating synaptic activity and acting as a neuromodulator and anti-excitatory buffer [62]. By contrast, OLZ had less pronounced effects on adenine metabolites, which could reflect lower energy expenditure due to suppression of arousal and GABAergic signaling. Interestingly, antipsychotics have been shown to affect mitochondrial function and oxidative stress, particularly under chronic exposure [63]. This may reflect an OLZ-induced impact on mitochondrial function or oxidative stress responses, potentially increasing reliance on nucleotide salvage pathways for ATP turnover [61].

Further supporting an altered energetic state, NADH levels were significantly reduced in the CPu of RIS-treated brains compared to CTRL and OLZ. This decrease may reflect enhanced mitochondrial NADH oxidation or diminished glycolytic activity, pointing to a shift in intracellular redox balance. Since NADPH, a related pyridine nucleotide, is essential for maintaining cellular antioxidant capacity *via* the glutathione and thioredoxin systems [64], we investigated broader metabolic shifts. Reflecting a potential downstream impact on these antioxidant pathways, we found a significant effect of both drugs on striatal levels of SAM, indicating reduced methylation potential, also related to COMT activity. Furthermore, these findings are consistent with previous studies showing that antipsychotic treatments, including OLZ, modulate lysophospholipid metabolism and influence phospholipase activity, both of which are integral to membrane remodeling and receptor signaling [8], [65].

It is important to note that the initial study was designed primarily to characterize the neuroPK profile of the drugs, using a 4-h intravenous infusion to ensure controlled and predictable systemic exposure [34], [35]. Given the remarkable regional and subregional transport differences observed, drug-target evaluations were subsequently performed. While this approach allowed preliminary insights into drug effects, the extended infusion duration likely limited the resolution of temporal pharmacodynamic relationships, particularly the identification of onset and peak responses. Consequently, interpretation of concentration–effect dynamics should be made with caution. Future studies employing multi-dose bolus administration or shorter infusion protocols may help refine these relationships and improve understanding of the compound's pharmacodynamic profile. Although pharmacokinetic steady state was achieved during the infusion, this should not be equated with the neuroadaptive state resulting from repeated or chronic antipsychotic treatment. The present findings therefore represent acute neurochemical and pharmacodynamic responses under controlled experimental exposure conditions. Acute exposure may produce metabolic responses that differ from the compensatory and neuroadaptive changes developing during chronic treatment, and extrapolation of these findings to long-term clinical treatment should therefore be made with caution. From this perspective, it should also be noted that, given the single–time point, unlabeled nature of the MSI data, the calculated [product]/[precursor] ratios should be interpreted as a semi-quantitative proxy for dopamine and purine catabolism, rather than a direct measure of compartment-specific metabolic rates. Nevertheless, it should also be mentioned that long-acting injectable formulations of second-generation antipsychotics have already been used in clinical practice, aiming at improving patients' compliance with the therapy [66]. These formulations include the intramuscular depot of olanzapine pamoate (e.g., Zyprexa Relprevv from Eli Lilly), with a half-life of approx. 30 days, and risperidone microspheres (e.g., Risperdal Consta) [66], [67]. The relatively small control group (n = 3) represents a limitation of the study, reducing statistical power and the precision of control-group variance estimates, thus future validation in prospectively powered studies with balanced group sizes is therefore warranted.

This study demonstrates that RIS and OLZ induce region-specific alterations in neurotransmitter metabolism, nucleotide homeostasis and oxidative stress regulation. RIS treatment was associated with increased levels of dopamine and norepinephrine downstream metabolites, alongside reductions in oxidative stress–related and nucleotide metabolites, consistent with altered catecholaminergic turnover and energy metabolism. In contrast, OLZ was associated with broader metabolic changes, including patterns consistent with altered mitochondrial function and increased oxidative stress. Complementary analysis of on-tissue tryptic peptides indicated potential, albeit limited, differences in cytoskeletal organization, synaptic processes, and mitochondrial-associated functions, supporting a link between metabolic alterations and cellular adaptations. Together, these findings suggest that antipsychotic drug exposure is associated with region-specific changes in neurochemical and metabolic pathways beyond receptor-level effects.

By resolving these alterations in a spatially defined manner, this study provides insight into region-specific neurochemical responses to antipsychotic treatment and highlights the value of spatially resolved metabolomic approaches for studying neuropharmacological effects *in situ*.

## Associated content

This manuscript is accompanied by supporting information.

## Author contributions

Y-R.L., N.D.: investigation, formal analysis, methodology, writing-original draft, writing-review and editing, and visualization. I.L., M.H.U.: conceptualization, methodology, editing, funding acquisition. R.S., A.N.: methodology, and editing. P.E.A.: conceptualization, methodology, resources, validation, editing, project administration, supervision, and funding acquisition as principal investigator. T.V.: conceptualization, methodology, resources, validation, editing, project administration, supervision. All authors have given approval to the final version of the manuscript.

## Declaration of interest

The authors declare the following competing financial interests: A.N., R.S., and P.E.A. are co-founders and shareholders of Tag-ON AB. The remaining authors declare no known competing financial interests.
