# Supplementary material for: Spatial pharmacometabolomic profiling of two antipsychotics reveals region-specific neurochemical alterations in the rat brain
Source: Neurotherapeutics. 2026 Oct 22;24(1):e01101. doi: 10.1016/j.neurot.2026.e01101 (PMC13718777; doi:10.1016/j.neurot.2026.e01101)
Supplement: Supplementary file 1 [file mmc1.pdf]

Supporting Information

Spatial Pharmacometabolomic Profiling of Two Antipsychotics Reveals Region-Specific Neurochemical Alterations in the Rat Brain

Yea-Rin Lee[1, #], Nivetha Dayanidhi[1, #], Irena Loryan[2], Margareta Hammarlund-Udenaes[2], Reza Shariatgorji[1], Anna Nilsson[1], Per E. Andrén[1,4] *, and Theodosia Vallianatou[1, *]

1. Department of Pharmaceutical Biosciences, Spatial Mass Spectrometry, Uppsala University, Uppsala SE-75124, Sweden
2. Department of Pharmacy, Translational PKPD Research Group, Uppsala University, Uppsala SE-75123, Sweden
3. Spatial Mass Spectrometry, Science for Life Laboratory, Uppsala University, Uppsala SE-75124, Sweden
4. Wits Integrated Molecular Physiology Research Initiative, Department of Physiology, School of Biomedical Sciences, Faculty of Health Sciences, University of the Witwatersrand, Johannesburg, South Africa

# First authors, Equal contribution

* Corresponding authors: per.andren@uu.se, theodosia.vallianatou@uu.se

Supplementary Tables

Table S1. Spray parameters for matrix and enzyme application in MALDI-MSI experiments.

Parameters	FMP-10 (matrix)	9-AA (matrix)	DHB (matrix)	Internal standards (RIS & OLZ)	Trypsin G (enzyme)	CHCA (matrix)
Sprayer	TM	TM	TM	TM	M3	M3
Concentration	1.8 mg/mL in 70% ACN	5 mg/mL in 80% MeOH	35 mg/mL in 50% ACN, 0.2% TFA	*30 ng/mL^{-1} (risperidone-d_4), and 40 ng/mL^{-1} (olanzapine-d_8) in 50% ACN, 0.2% TFA	0.1 µg/µL trypsin gold in milli-Q water	7 mg/mL CHCA in 50% ACN, 0.1% TFA
Temperature (°C)	90	75	95	90	45	77
Flow rate (µL/min)	80	70	70	70	20	70
Pressure (psi)	6	6	6	6	9	9
Velocity (mm/min)	1100	1100	1200	1100	1200	1100
Track spacing (mm)	2	2	3	2	2.5	2.5
Number of passes	20	6	8	6	15	10

Abbreviations: ACN: Acetonitrile, MeOH: Methanol, TFA: Trifluoroacetic Acid, and CHCA: α-Cyano-4-hydroxycinnamic Acid*Applied only when the CASI experiment was performed for visualization of OLZ and RIS at hippocampal brain level.

Table S2. Instrument settings for MALDI analysis.

**Parameters**	**FMP-10: Neurotransmitters**	**9-AA: Small molecules (metabolites)**	**DHB: Small molecules (metabolites)**	**Trypsin & CHCA: Tryptic peptides**
		FTICR		
Scan begin	80 *m/z*	80 *m/z*	80 *m/z*	NA
Scan end	1000 *m/z*	1000 *m/z*	1000 *m/z*	NA
Ion polarity	Positive	Negative	Positive	NA
Scan mode	Quadrature phase detection (QPD) (2ω)	Quadrature phase detection (QPD) (2ω)	Quadrature phase detection (QPD) (2ω)	NA
Shots	60-100 µm	60-100 µm	60-100 µm	NA
Size	Medium	Medium	Medium	NA
Power	Optimized prior to acquisition	Optimized prior to acquisition	Optimized prior to acquisition	NA
Q1 isolation	379 *m/z*	120 *m/z*	250 *m/z*	NA
Frequency	4 MHz	4 MHz	4 MHz	NA
Lock mass	555.2231 (matrix cluster ion)	193.0771 (matrix cluster ion)	153.0339 (matrix cluster ion)	NA
		TimsTOF fleX		
Scan begin	80 *m/z*	80 *m/z*	80 *m/z*	700.0 *m/z*
Scan end	1600 *m/z*	1600 *m/z*	1600 *m/z*	3200.0 *m/z*
Ion polarity	Positive	Negative	Positive	Positive
Scan mode	MS	MS	MS	MS
Shots	100	100	100	200
Frequency	10 kHz	10 kHz	10 kHz	10 kHz
Power	58%	58%	58%	60%
Laser	30, 30 µm (x, y)	30, 30 µm (x, y)	30, 30 µm (x, y)	50, 50 µm (x, y)
Raster	30, 30 µm	30, 30 µm	30, 30 µm	50, 50 µm

Table S3. Evaluation of the PLS-DA models using CV-ANOVA.

Matrix	p-value (striatal level)	F-statistic (striatal level)	p-value (hippocampal level)	F-statistic (hippocampal level)
FMP-10	0.0412259	2.64538	0.00057394	5.95359
DHB	0.00748416	3.92843	0.0172274	3.17217
9AA	0.0132344	3.48516	0.0341962	2.69978

Table S4. Identification of significant *m/z* values of neurotransmitters and metabolites across CTRL, RIS, and OLZ groups in the striatal and hippocampal levels.

Brain level	Matrix	Exper m/z	Theor m/z	Mass error (ppm)	Name	Formula	Adduct	Identification
Striatum	FMP-10	450.1705	450.1700	1.11	HVA (Homovanillic Acid)	C9H10O4	M+1FMP-10	MS/MS [5, 6]
Striatum	FMP-10	689.2440	689.2435	0.73	DOPAC (3,4-Dihydroxyphenylacetic Acid)	C8H8O4	M+2FMP-10-CH3	MS/MS [6, 7]
Striatum	FMP-10	435.2071	435.2067	0.92	3MT (3-methoxytyramine)	C9H13NO2	M+1FMP-10	MS/MS [6]
Hippocampus	FMP-10	690.2747	690.2751	-0.60	NE (Norepinephrine)	C8H11NO3	M+2FMP-10-CH3	MS/MS [5, 6]
Hippocampus	FMP-10	691.2600	691.2591	1.25	DOPEG (3,4-Dihydroxyphenylethylene glycol)	C8H10O4	M+2FMP-10-CH3	MS/MS [5, 6]
Hippocampus	FMP-10	371.1760	371.1754	1.62	GABA (γ-Aminobutyric Acid)	C4H9NO2	M+1FMP-10	MS/MS [5, 6]
Hippocampus	FMP-10	404.1511	404.1506	1.24	Hypoxanthine	C5H4N4O	M+1FMP-10	MS/MS [6]
Striatum	DHB	268.1040	268.1040	0.00	Adenosine	C10H13N5O4	M+H	MS/MS
Striatum	DHB	465.9926	465.9927	0.21	ADP (Adenosine diphosphate)	C10H15N5O10P2	M+K	MS/MS
Striatum	DHB	583.9148	583.9146	0.34	ATP (Adenosine triphosphate)	C10H16N5O13P3	M+2K-H	Mass match and multiple adduct cross-validation
Striatum	DHB	742.0444	742.0439	0.67	NADH (Nicotinamide adenine dinucleotide, reduced form)	C21H28N7O14P2	M+2K	Mass match
Hippocampus	DHB	465.9926	465.9927	-0.21	ADP (Adenosine diphosphate)	C10H15N5O10P2	M+K	MS/MS
Hippocampus	DHB	583.9148	583.9146	0.34	ATP (Adenosine triphosphate)	C10H16N5O13P3	M+2K-H	Mass match and multiple adduct cross-validation
Striatum	9-AA	263.0231	263.0232	-0.38	MHPG-SO_4 (3-methoxy-4-hydroxyphenylethyleneglycol sulfate)	C9H12O7S	M-H	MS/MS
Striatum	9-AA	619.2889	619.2880	1.45	LysoPI (20:4/0:0) (Lysophosphatidylinositol)	C29H49O12P	M-H	Mass match [8]
Hippocampus	9-AA	263.0231	263.0232	-0.38	MHPG-SO_4 (3-methoxy-4-hydroxyphenylethyleneglycol sulfate)	C9H12O7S	M-H	MS/MS
Hippocampus	9-AA	368.9993	368.9992	0.27	SBP (Sedoheptulose 1,7-bisphosphate)	C7H16O13P2	M-H	Mass match and multiple adduct cross-validation
Hippocampus	9-AA	426.0221	426.0218	0.70	ADP (Adenosine diphosphate)	C10H15N5O10P2	M-H	MS/MS
Hippocampus	9-AA	619.2889	619.2880	1.45	LysoPI (20:4/0:0) (Lysophosphatidylinositol)	C29H49O12P	M-H	Mass match [8]

Table S5. Detection of significant metabolites using untargeted one-way ANONA

Brain region	Matrix	Exper m/z	Name	FDR	Post-hoc Fisher's LSD
CPu	FMP-10	450.1705	HVA (Homovanillic Acid)	*0.19378/ 0.011688	OLZ - CTRL (p=0.000865); RIS - CTRL (p=0.000118)
CPu	FMP-10	689.2440	DOPAC (3,4-Dihydroxyphenylacetic Acid)	*0.4343/ 0.15648	RIS - CTRL (p=0.00522)
CPu	FMP-10	435.2071	3MT (3-methoxytyramine)	*0.19378/0.012359	OLZ - CTRL (p=0.00352); OLZ - RIS (p=0.000256)
CTX-hippocampal level	FMP-10	371.1760	GABA (γ-Aminobutyric Acid)	*0.020124/0.0021429	CTRL - OLZ (p=4.1e-05); CTRL - RIS (p=0.0137); RIS - OLZ (p=0.00134)
CTX-striatal level	FMP-10	690.2747	NE (Norepinephrine)	*0.033009/0.016941	CTRL - RIS (p=3e-04); OLZ - RIS (p=0.000186)
CTX-hippocampal level	FMP-10	690.2747	NE (Norepinephrine)	*0.011007/0.0012749	OLZ - CTRL (p=0.0159); CTRL - RIS (p=0.00548); OLZ - RIS (p=7e-06)
CTX-hippocampal level	FMP-10	691.260	DOPEG (3,4-Dihydroxyphenylethylene glycol)	*0.058851/0.017743	CTRL - OLZ (p=0.00147); CTRL - RIS (p=0.0421); RIS - OLZ (p=0.0449)
CTX-hippocampal level	FMP-10	404.1511	Hypoxanthine	0.036509	CTRL - OLZ (p=0.000401); CTRL - RIS (p=0.0435); RIS - OLZ (p=0.00804)
CPu	DHB	268.1040	Adenosine	0.0070115	RIS - CTRL (p=0.000156); RIS - OLZ (p=0.000127)
CTX-striatal level	DHB	465.9926	ADP (Adenosine diphosphate)	4.4406e-06	CTRL - OLZ (p=0.0317); CTRL - RIS (p<1e-06); OLZ - RIS (p<1e-06)
CTX-hippocampal level	DHB	465.9926	ADP (Adenosine diphosphate)	0.016181	CTRL - RIS (p=0.000112); OLZ - RIS (p=0.000398)
CPu	DHB	465.9926	ADP (Adenosine diphosphate)	0.00096699	CTRL - OLZ (p=0.0102); CTRL - RIS (p=3e-06); OLZ - RIS (p=1.5e-05)
CTX-striatal level	DHB	583.9148	ATP (Adenosine triphosphate)	3.1978e-05	CTRL - OLZ (p=0.02); CTRL - RIS (p<1e-06); OLZ - RIS (p=1e-06)
CTX-hippocampal level	DHB	583.9148	ATP (Adenosine triphosphate)	0.0086716	CTRL - OLZ (p=0.0469); CTRL - RIS (p=1.3e-05); OLZ - RIS (p=8.8e-05)
Hip	DHB	583.9148	ATP (Adenosine triphosphate)	0.010452	CTRL - OLZ (p=0.032); CTRL - RIS (p=7e-06); OLZ - RIS (p=6e-05)
CPu	DHB	583.9148	ATP (Adenosine triphosphate)	0.00015166	CTRL - OLZ (p=0.000343); CTRL - RIS (p<1e-06); OLZ - RIS (p<1e-06)
CPu	DHB	742.0444	NADH (Nicotinamide adenine dinucleotide, reduced form)	0.077099	CTRL - RIS (p=0.00166); OLZ - RIS (p=0.0169)
CTX-striatal level	9-AA	263.0231	MHPG-SO_4 (3-methoxy-4-hydroxyphenylethyleneglycol sulfate)	0.0032102	RIS - CTRL (p=4e-06); RIS - OLZ (p=3e-06)
CTX-hippocampal level	9-AA	263.0231	MHPG-SO_4 (3-methoxy-4-hydroxyphenylethyleneglycol sulfate)	1.4619e-05	OLZ - CTRL (p=0.0104); RIS - CTRL (p<1e-06); RIS - OLZ (p<1e-06)
Hip	9-AA	263.0231	MHPG-SO_4 (3-methoxy-4-hydroxyphenylethyleneglycol sulfate)	0.00025114	OLZ - CTRL (p=0.00582); RIS - CTRL (p<1e-06); RIS - OLZ (p=1e-06
CTX-striatal level	9-AA	619.2889	LysoPI (20:4/0:0) (Lysophosphatidylinositol)	0.030739	OLZ - CTRL (p=0.0243); OLZ - RIS (p=0.00013)
CTX-hippocampal level	9-AA	619.2889	LysoPI (20:4/0:0) (Lysophosphatidylinositol)	0.018637	OLZ - CTRL (p=0.0011); OLZ - RIS (p=2.7e-05)
Hip	9-AA	368.9993	SBP (Sedoheptulose 1,7-bisphosphate)	0.032066	CTRL - RIS (p=0.0115); OLZ - RIS (p=0.000126)

* FDR value from untargeted FMP-10 data (2226 features) / FDR value from targeted FMP-10 data (60 features)

Table S6. List of peptides (proteins) detected by MALDI-MSI and matched to LC-MS/MS data within a ±10 ppm mass tolerance. Peptide sequences and database entries were obtained from the UniProt Knowledgebase (www.uniprot.org), restricted to *Rattus norvegicus* and filtered for reviewed Swiss-Prot entries (Swiss Institute of Bioinformatics, Switzerland).

MALDI *m/z*	LC-MS/MS *m/z*	Error (ppm)	Entry	Gene	Protein	Sequence
976.455	976.448	7.54	ACTB_RAT	Actb	Actin, cytoplasmic 1	AGFAGDDAPR
			ACTG_RAT	Actg	Actin, cytoplasmic 2	AGFAGDDAPR
988.514	988.505	8.64	CLCA_RAT	Clta	Clathrin light chain A	LEALDANSR
1032.601	1032.594	6.55	H33_RAT	H3f3b	Histone H3.3	YRPGTVALR
1070.539	1070.537	1.68	PLPP_RAT	Plpp	Chronophin	LTEACAHLR
1074.559	1074.557	1.47	AMPH_RAT	Amph	Amphiphysin	VGFYVNTFK
1082.556	1082.554	1.94	DYN1_RAT	Dnm1	Dynamin-1	YMLSVDLNK
1084.567	1084.567	0.52	PDIA3_RAT	Pdia3	Protein disulfide-isomerase A3	YGVSGYPLTK
1089.489	1089.495	-6.20	CLCB_RAT	Cltb	Clathrin light chain B	DLEEWNQR
1100.563	1100.569	-5.85	HOME3_RAT	Homer3	Homer protein homolog 3	NAELEQQLR
1103.526	1103.532	-5.87	GFAP_RAT	Gfap	Glial fibrillary acidic protein	QEADEATLAR
1121.580	1121.583	-2.85	NFM_RAT	Nefm	Neurofilament medium polypeptide	EYQDLLNVK
			NFH_RAT	Nefh	Neurofilament heavy polypeptide	EYQDLLNVK
			NFL_RAT	Nefl	Neurofilament light polypeptide	EYQDLLNVK
			AINX_RAT	Ina	Alpha-internexin	EYQDLLNVK
1127.563	1127.568	-4.58	REEP5_RAT	Reep5	Receptor expression-enhancing protein 5	HESQVDSVVK
1132.529	1132.526	2.01	ACTB_RAT	Actb	Actin, cytoplasmic 1	GYSFTTTAER
			ACTG_RAT	Actg	Actin, cytoplasmic 2	GYSFTTTAER
1133.548	1133.537	9.60	SFXN5_RAT	Sfxn5	Sideroflexin-5	FQQTSFYGR
1138.571	1138.573	-1.70	SPTN1_RAT	Sptan1	Spectrin alpha chain, non-erythrocytic 1	LLVSSEDYGR
1143.637	1143.634	2.68	TBB5_RAT	Tubb5	Tubulin beta-5 chain	LAVNMVPFPR
			TBB2A_RAT	Tubb2a	Tubulin beta-2A chain	LAVNMVPFPR
			TBB2B_RAT	Tubb2b	Tubulin beta-2B chain	LAVNMVPFPR
			TBB3_RAT	Tubb3	Tubulin beta-3 chain	LAVNMVPFPR
			TBB4B_RAT	Tubb4b	Tubulin beta-4B chain	LAVNMVPFPR
1147.474	1147.464	8.84	1433E_RAT	Ywhae	14-3-3 protein epsilon	YDEMVESMK
1153.566	1153.573	-5.67	DCE1_RAT	Gad1	Glutamate decarboxylase 1	ANSVTWNPHK
			DCE2_RAT	Gad2	Glutamate decarboxylase 2	ANSVTWNPHK
1183.608	1183.613	-4.80	NCAM1_RAT	Ncam1	Neural cell adhesion molecule 1	LPSGSDHVMLK
1190.585	1190.589	-3.19	PDIA4_RAT	Pdia4	Protein disulfide-isomerase A4	VDATEQTDLAK
1191.581	1191.582	-1.11	CLCB_RAT	Cltb	Clathrin light chain B	VAQLCDFNPK
1198.710	1198.705	4.49	ACTB_RAT	Actb	Actin, cytoplasmic 1	AVFPSLVGRPR
			ACTG_RAT	Actg	Actin, cytoplasmic 2	AVFPSLVGRPR
1213.564	1213.562	1.43	BSN_RAT	Bsn	Protein bassoon	QAAEMEELHR
1220.691	1220.688	2.75	GRIA2_RAT	Gria2	Glutamate receptor 2	SLFQDLELKK

1251.617	1251.619	-1.20	LMNB1_RAT	Lmnb1	Lamin-B1	ALYETELADAR
1258.690	1258.690	0.05	TBB5_RAT	Tubb5	Tubulin beta-5 chain	FPGQLNADLRK
			TBB2A_RAT	Tubb2a	Tubulin beta-2A chain	FPGQLNADLRK
			TBB2B_RAT	Tubb2b	Tubulin beta-2B chain	FPGQLNADLRK
			TBB3_RAT	Tubb3	Tubulin beta-3 chain	FPGQLNADLRK
			TBB4B_RAT	Tubb4b	Tubulin beta-4B chain	FPGQLNADLRK
1274.731	1274.725	4.99	HBB1_RAT	Hbb	Hemoglobin subunit beta-1	LLVVYPWTQR
1295.685	1295.695	-7.39	HSP74_RAT	Hspa4	Heat shock 70 kDa protein 4	QDLPALEEKPR
1309.665	1309.678	-9.91	COF1_RAT	Cfl1	Cofilin-1	AVLFCLSEDKK
1322.696	1322.694	1.26	1433E_RAT	Ywhae	14-3-3 protein epsilon	KEAAENSLVAYK
1324.588	1324.594	-5.01	MAP1A_RAT	Map1a	Microtubule-associated protein 1A	SAPCGSLAFSGDR
1336.637	1336.631	4.49	MBP_RAT	Mbp	Myelin basic protein	YLATASTMDHAR
1385.737	1385.726	7.49	CTNB1_RAT	Ctnnb1	Catenin beta-1	LLNDEDQVVVNK
1409.734	1409.742	-5.49	GFAP_RAT	Gfap	Glial fibrillary acidic protein	VDFSLAGALNAGFK
1412.725	1412.716	6.47	SYT1_RAT	Syt1	Synaptotagmin-1	VFVGYNSTGAELR
1416.733	1416.736	-2.27	CK068_RAT	Bles03	UPF0696 protein C11orf68 homolog	TTPATELDAWLAK
1432.760	1432.761	-0.75	AT1A2_RAT	Atp1a2	Sodium/potassium-transporting ATPase subunit alpha-2	VLGFCQLNLPSGK
1460.716	1460.716	-0.38	MBP_RAT	Mbp	Myelin basic protein	TQDENPVVHFFK
1493.705	1493.711	-4.04	RAB14_RAT	Rab14	Ras-related protein Rab-14	TGENVEDAFLEAAK
1494.710	1494.722	-7.75	S2512_RAT	Slc25a12	Electrogenic aspartate/glutamate antiporter SLC25A12, mitochondrial	YLGLYNDPNSNPK
1498.674	1498.684	-6.59	SPTN2_RAT	Sptbn2	Spectrin beta chain, non-erythrocytic 2	MLTAQDVSYDEAR
1504.686	1504.684	1.21	HNRH1_RAT	Hnrnph1	Heterogeneous nuclear ribonucleoprotein H	GLPWSCSADEVQR
1539.797	1539.791	3.82	TPIS_RAT	Tpi1	Triosephosphate isomerase	DLGATWVVLGHSER
1547.659	1547.650	5.84	ELOC_RAT	Eloc	Elongin-C	TYGGCEGPDAMYVK
1548.720	1548.713	4.53	1433Z_RAT	Ywhaz	14-3-3 protein zeta/delta	SVTEQGAELSNEER
1566.759	1566.744	9.68	EAA4_RAT	Eaat4	Excitatory amino acid transporter 4	NMFPPNLVEACFK
			EAA1_RAT	Eaat1	Excitatory amino acid transporter 1	NMFPPNLVEACFK
1665.734	1665.742	-4.65	C1QBP_RAT	C1qbp	Complement component 1 Q subcomponent-binding protein, mitochondrial	MSGDWELEVNGTEAK

Table S7. Statistical analysis of peptides (proteins) across CTRL, RIS and OLZ groups. Proteins are ranked based on FDR-corrected *p*-values, with higher-ranked entries indicating stronger evidence for group differences. None of the peptides (proteins) reached statistical significance following correction for multiple comparisons.

F_statistic[#]	*p*_value[&]	MALDI *m/z*	Gene	p_value_FDR[%]	Significant_FDR_5%*
5.08	0.0253	1220.691	Gria2	0.5941	No
5.73	0.0179	1147.474	Ywhae	0.5941	No
3.87	0.0504	1539.797	Tpi1	0.6842	No
3.64	0.0582	1416.733	Bles03	0.6842	No
2.81	0.0998	1191.581	Cltb	0.7934	No
2.79	0.1013	1385.737	Ctnnb1	0.7934	No
2	0.1774	1498.674	Sptbn2	0.8963	No
1.73	0.2187	1084.567	Pdia3	0.8963	No
1.49	0.2647	1100.563	Homer3	0.8963	No
2.06	0.1703	1493.705	Rab14	0.8963	No
1.61	0.2393	1213.564	Bsn	0.8963	No
1.73	0.2191	1198.710	Actb & Actg	0.8963	No
1.98	0.1804	1295.685	Hspa4	0.8963	No
1.48	0.267	1322.696	Ywhae	0.8963	No
0.6	0.5662	1504.686	Hnrnph1	0.9724	No
0.08	0.9246	1309.665	Cfl1	0.9724	No
0.3	0.7428	1324.588	Map1a	0.9724	No
0.59	0.5685	1409.734	Gfap	0.9724	No
1.08	0.3705	1548.720	Ywhaz	0.9724	No
0.07	0.931	1547.659	Eloc	0.9724	No
0.88	0.4383	1412.725	Syt1	0.9724	No
0.1	0.9088	1432.760	Atp1a2	0.9724	No
0.6	0.5632	1460.716	Mbp	0.9724	No
1.05	0.3813	1494.710	Slc25a12	0.9724	No
0.75	0.4931	1336.637	Mbp	0.9724	No
0.1	0.9085	1274.731	Hbb	0.9724	No
0.43	0.6616	976.455	Actb & Actg	0.9724	No
0.61	0.5607	1251.617	Lmnb1	0.9724	No
0.52	0.6097	988.514	Clta	0.9724	No
0.29	0.7541	1032.601	H3f3b	0.9724	No
0.39	0.6878	1070.539	Plpp	0.9724	No
0.57	0.5815	1082.556	Dnm1	0.9724	No
0.2	0.8219	1089.489	Cltb	0.9724	No
0.2	0.8237	1103.526	Gfap	0.9724	No
0.31	0.7396	1121.580	Nefm, Nefh, Nefl & Ina	0.9724	No
0.1	0.9024	1258.690	Tubb5, Tubb2a, Tubb2b, Tubb3 & Tubb4b	0.9724	No
0.36	0.7034	1127.563	Reep5	0.9724	No
0.34	0.716	1133.548	Sfxn5	0.9724	No

1.15	0.3503	1138.571	Sptan1	0.9724	No
0.39	0.6881	1153.566	Gad1 & Gad2	0.9724	No
0.6	0.563	1183.608	Ncam1	0.9724	No
0.23	0.8008	1190.585	Pdia4	0.9724	No
0.43	0.6591	1566.759	Eaat4 & Eaat1	0.9724	No
0.17	0.8489	1132.529	Actb & Actg	0.9724	No
0.4	0.681	1665.734	C1qbp	0.9724	No
0.03	0.9718	1143.637	Tubb5, Tubb2a, Tubb2b, Tubb3 & Tubb4b	0.993	No
0.01	0.9941	1074.559	Amph	0.9941	No

The F-value from the ANOVA test: Indicates the ratio of variance between groups to variance within groups. Higher F-values suggest larger differences between groups.

& The uncorrected p-value from the ANOVA test: Represents the raw statistical significance before any adjustment for multiple comparisons.

% The p-value after False Discovery Rate (FDR) correction: Adjusted p-value accounting for the multiple tests conducted (47 tests in total). This reduces the likelihood of false positives.

* Simple Yes/No indicator: Denotes whether the FDR-corrected p-value is below the significance threshold of 0.05.

Supplementary Figures

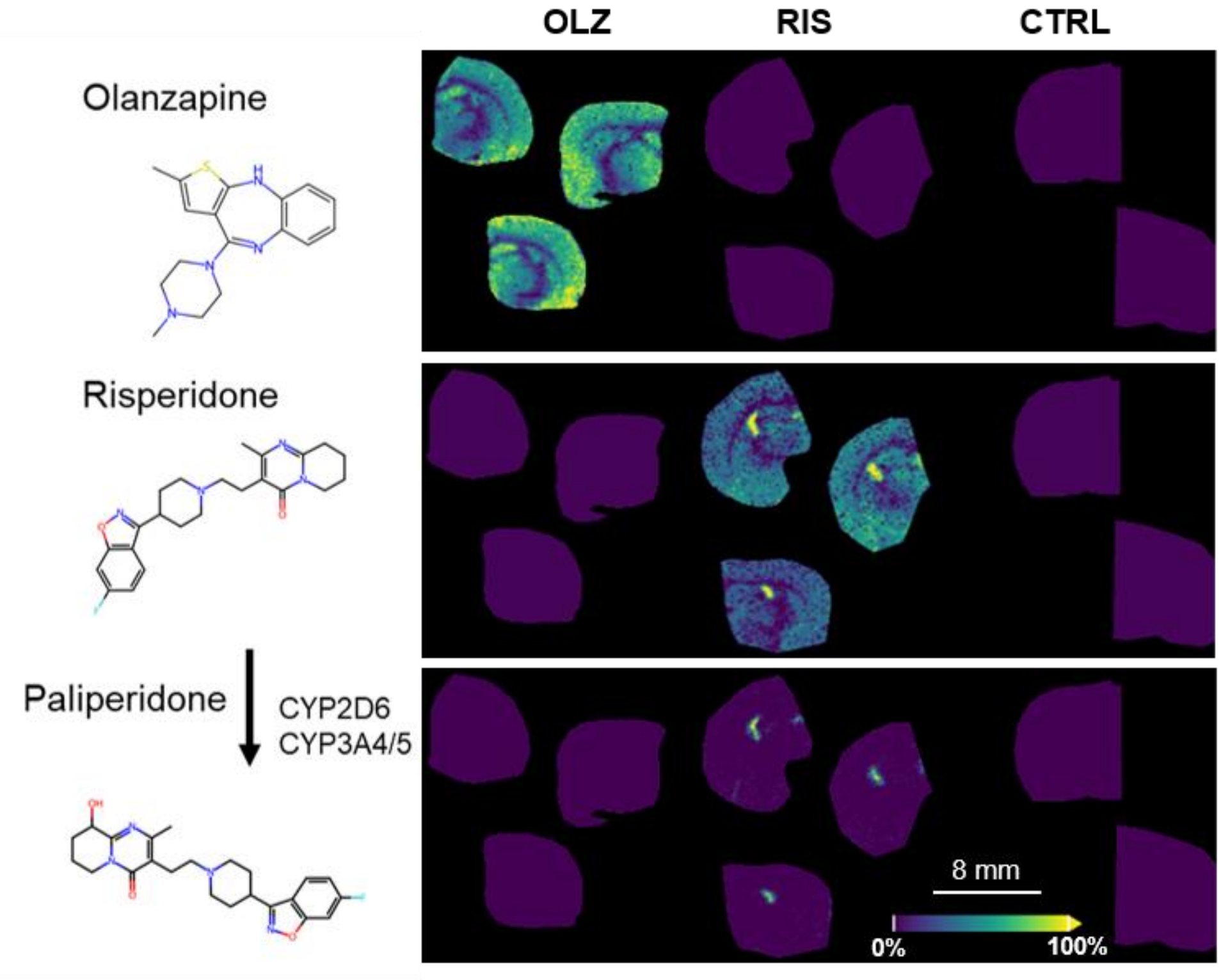

Figure S1. Representative MALDI-MSI ion images illustrating the localization of antipsychotic drugs across CTRL, RIS, and OLZ groups. Ion images show the spatial distribution of risperidone, paliperidone, and olanzapine. Notably, the active metabolite of RIS, paliperidone (9-hydroxyrisperidone), was confirmed *via* MALDI-MSI for the first time in tissue. Data were collected in positive ionization mode using DHB as the MALDI matrix.

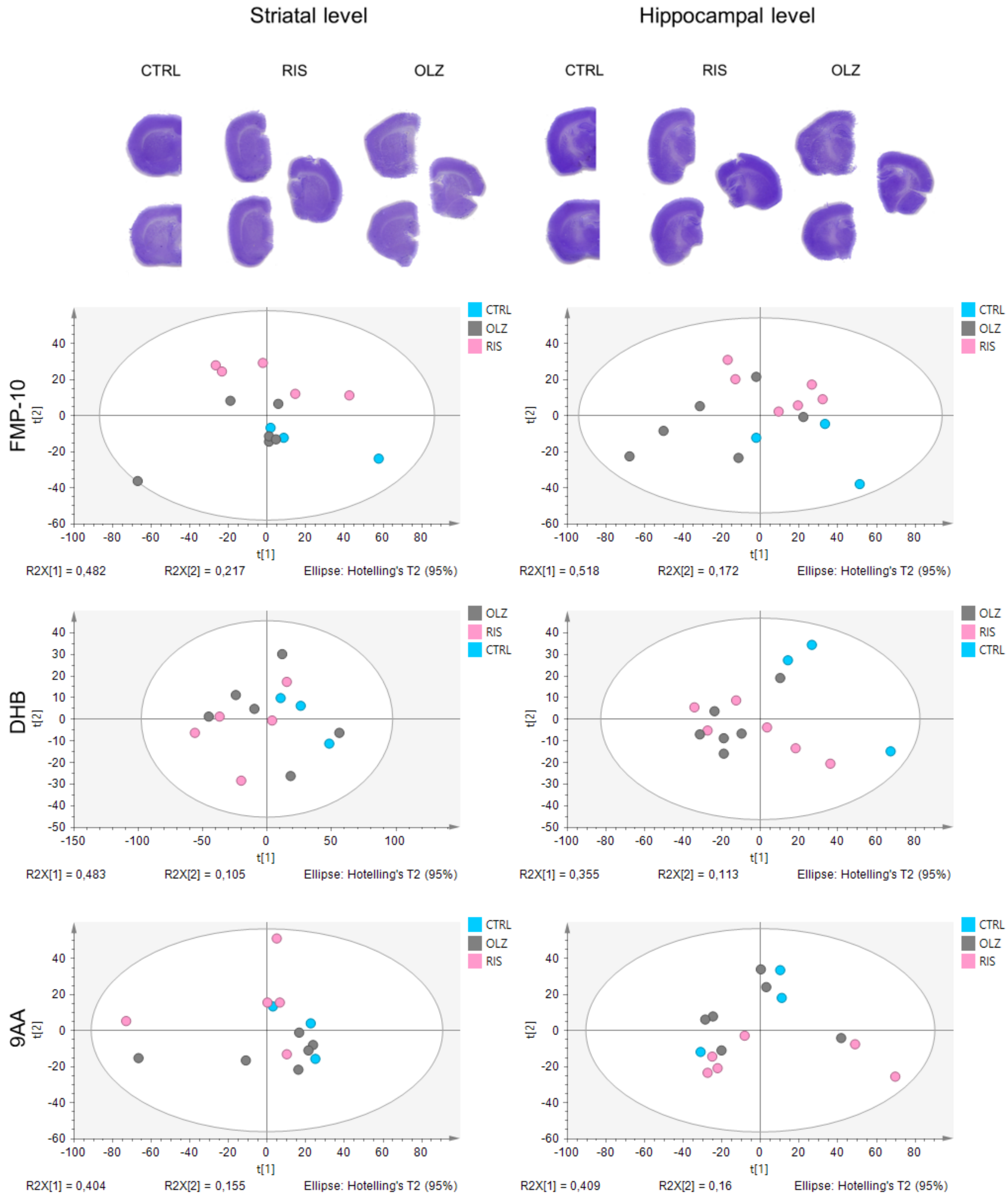

Figure S2. Representative Nissl-stained tissue sections and PCA-based assessment of group clustering. Nissl-stained sections illustrate annotated brain regions at the striatal and hippocampal levels, corresponding to regions analyzed by MALDI-MSI. PCA was performed for each dataset (FMP-10, DHB, and 9-AA) to evaluate group clustering and identify potential outliers.

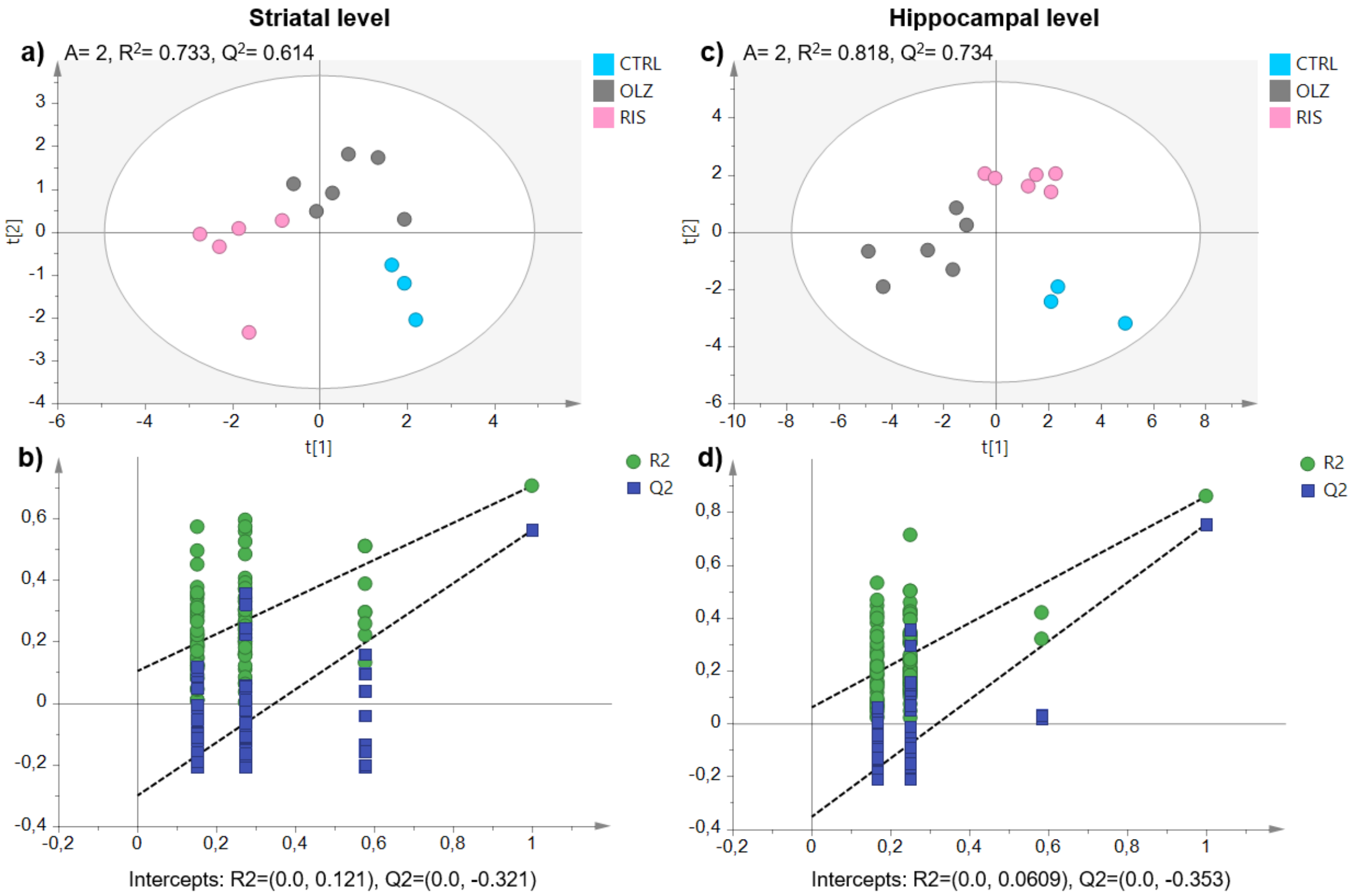

Figure S3. PLS-DA results and validation using the FMP-10 reactive matrix on whole brain tissue level. (a) Scores plot and corresponding statistics of the optimized PLS-DA model on the striatal level. (b) Permutation tests plot (100 permutations) of the PLS-DA model on the striatal level. The derived R^2 and Q^2 values are significantly lower than the original model indicating model robustness. (c) Scores plot and corresponding statistics of the optimized PLS-DA model on the hippocampal level. (d) Permutation tests plot (100 permutations) of the PLS-DA model on the hippocampal level. The derived R^2 and Q^2 values are significantly lower than the original model indicating model robustness. A, number of principal components.

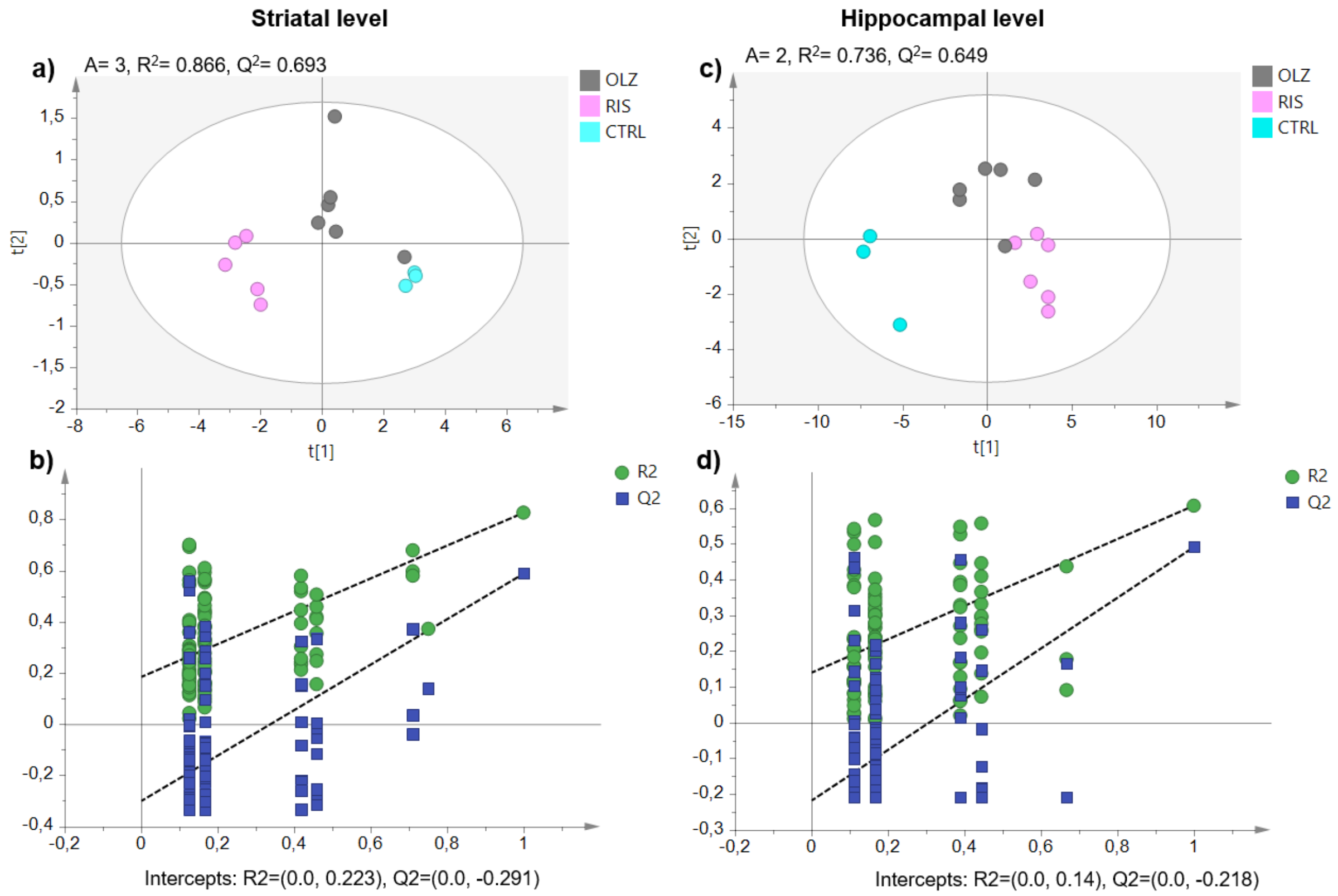

Figure S4. PLS-DA results and validation using DHB as matrix on whole brain tissue level. (a) Scores plot and corresponding statistics of the optimized PLS-DA model on the striatal level. (b) Permutation tests plot (100 permutations) of the PLS-DA model on the striatal level. The derived R^2 and Q^2 values are significantly lower than the original model indicating model robustness. (c) Scores plot and corresponding statistics of the optimized PLS-DA model on the hippocampal level. (d) Permutation tests plot (100 permutations) of the PLS-DA model on the hippocampal level. The derived R^2 and Q^2 values are significantly lower than the original model indicating model robustness. A, number of principal components.

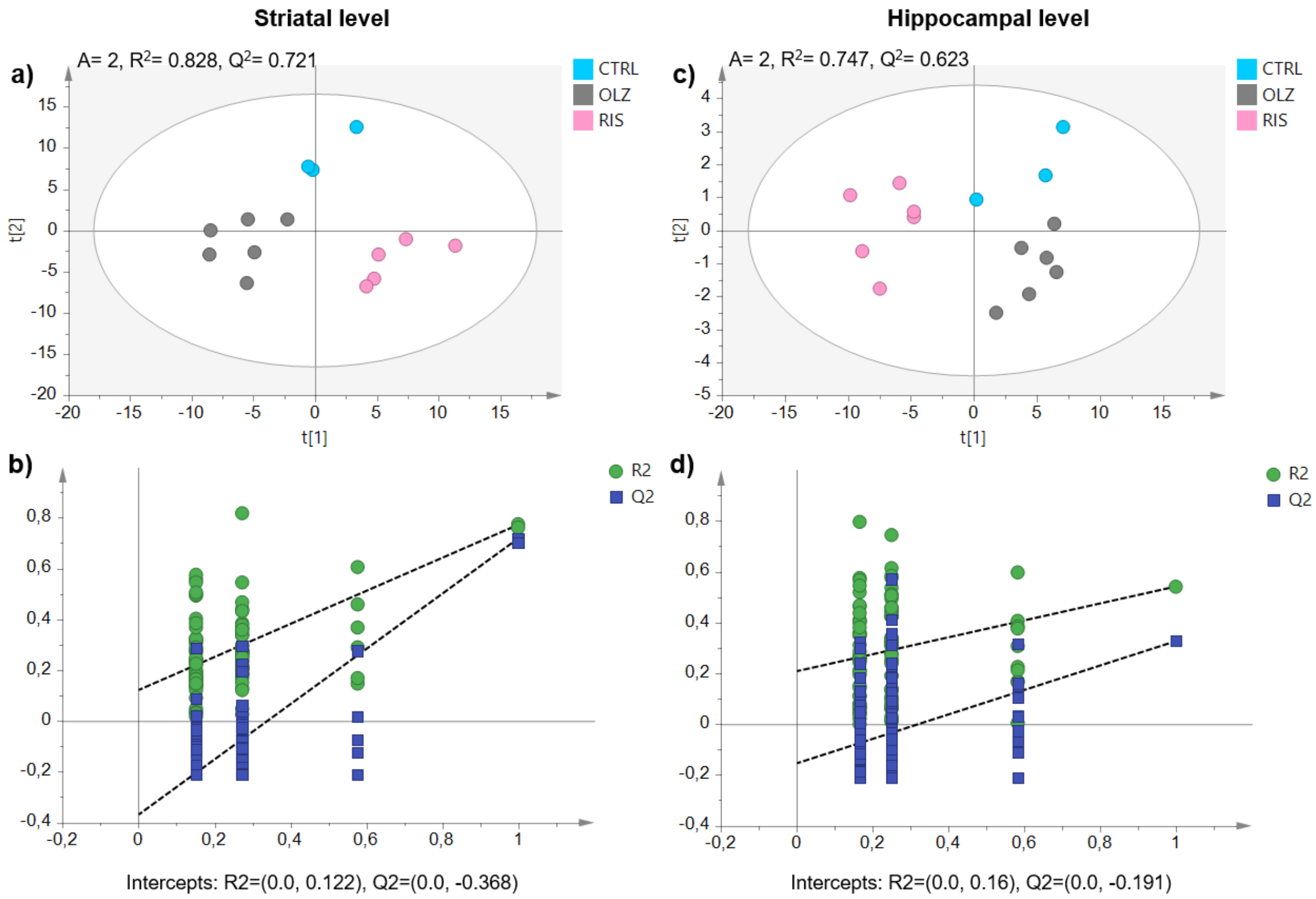

Figure S5. PLS-DA results and validation using 9AA as matrix on whole brain tissue level. (a) Scores plot and corresponding statistics of the optimized PLS-DA model on the striatal level. (b) Permutation tests plot (100 permutations) of the PLS-DA model on the striatal level. The derived R^2 and Q^2 values are significantly lower than the original model indicating model robustness. (c) Scores plot and corresponding statistics of the optimized PLS-DA model on the hippocampal level. (d) Permutation tests plot (100 permutations) of the PLS-DA model on the hippocampal level. The derived R^2 and Q^2 values are significantly lower than the original model indicating model robustness. A, number of principal components.

a)

Theoretical *m/z*	Experimental *m/z*	UniProt_ID	Sequence	Difference	Mean	Difference_ppm
976.448	976.448	ACTB_RAT	AGFAGDDAPR	0	976.448	0
1198.706	1198.705	ACTB_RAT	AVFPSIVGRPR	-0.001	1198.706	-0.83423
1336.631	1336.631	MBP_RAT	YLATASTMDHAR	0	1336.631	0
1460.717	1460.716	MBP_RAT	TQDENPVVHFFK	-0.001	1460.717	-0.6846

b)

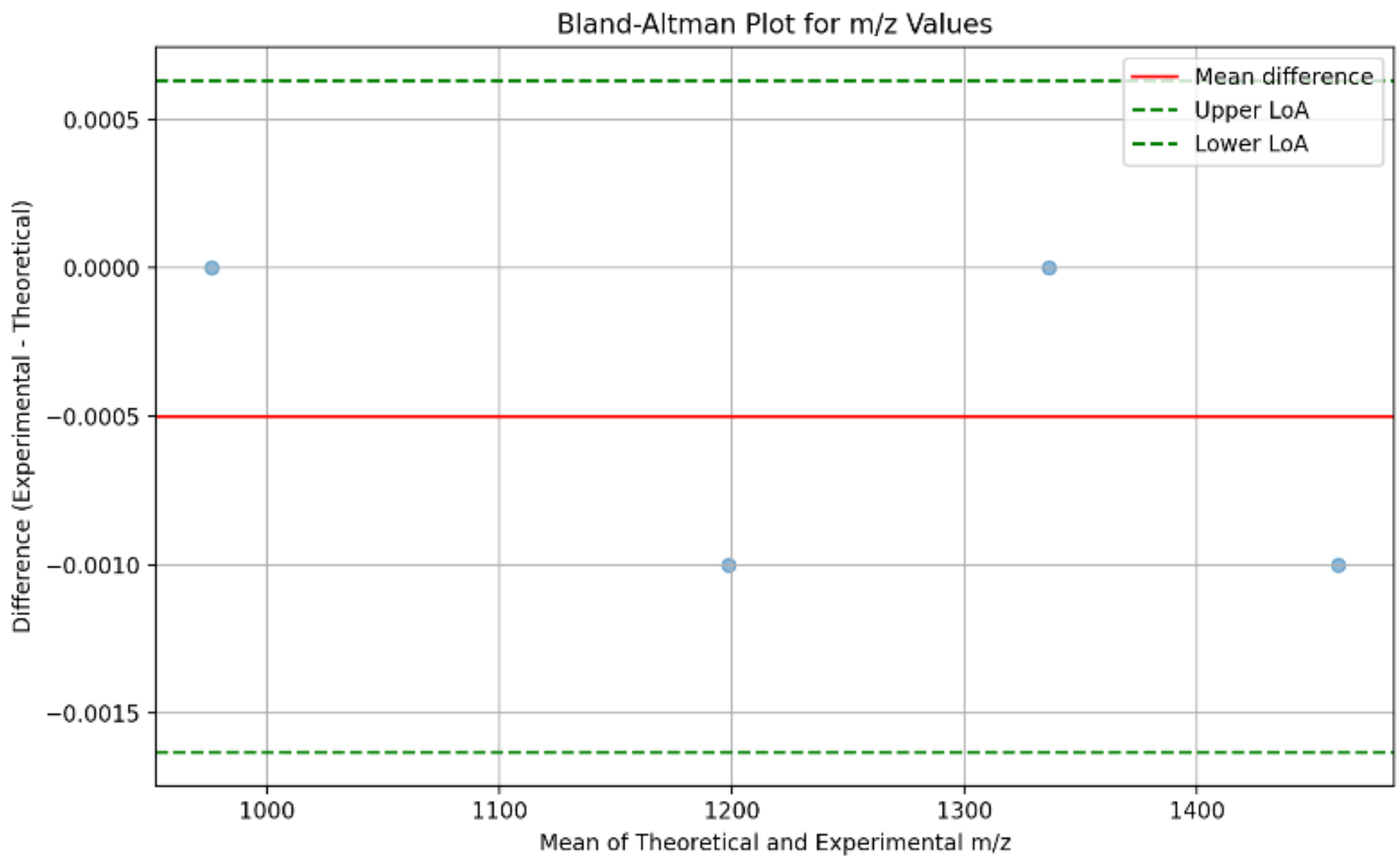

Figure S6. Validation of peptide identification through comparison of experimental and theoretical *m/z* values. a) Side-by-side comparison of experimental *m/z* values obtained from LC-MS/MS with corresponding theoretical *m/z* values for selected peptides—976.4 and 1198.7 for actin, and 1336.6 and 1460.7 for myelin basic protein (MBP). These peptides (proteins) were selected based on their well-established identification in previous studies, providing reliable references for validation[9-12]. b) Bland–Altman plot illustrating the percentage differences between theoretical and experimental *m/z* values for each peptide. This plot evaluates the agreement between observed data and theoretical expectations, identifying potential systematic deviations.

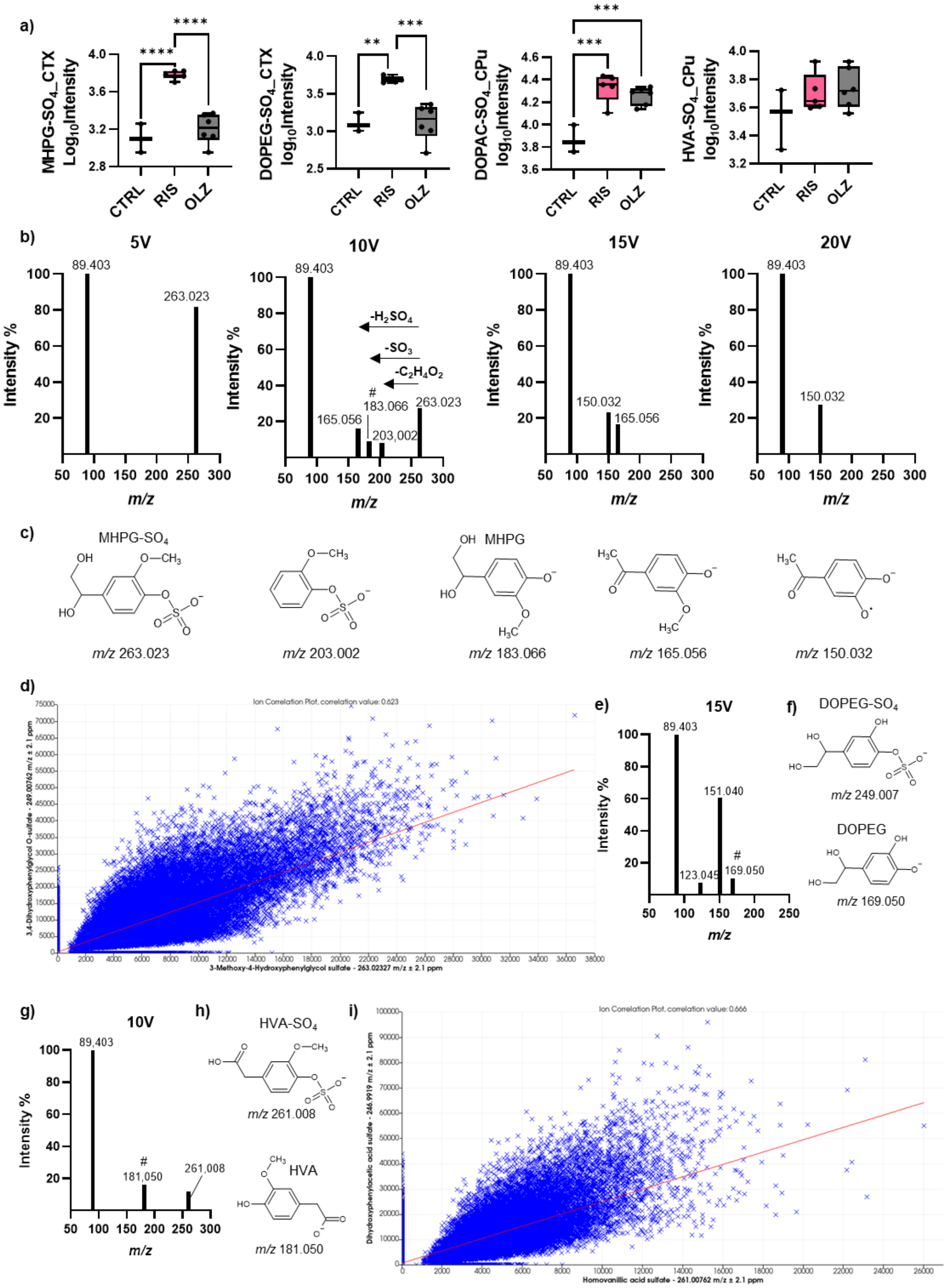

Figure S7. Regional antipsychotics induced effects on and structural validation of sulphated catecholaminergic metabolites. (a) Representative box plots showing log10-normalized intensity of MHPG-SO_4, DOPEG-SO_4, DOPAC-SO_4 and HVA-SO_4 in the CTX and Hip. Box plots indicate statistically significant group differences, presented by asterisks: * $p \leq 0.05$, and ** $p \leq 0.01$, ***$p \leq 0.001$, ****$p \leq 0.0001$. (b) Tandem MS spectra (*m/z* values

extracted at 1.5 signal-to-noise ratio) collected from rat brain tissue sections (5V-10V) for metabolite identification of MHPG-SO_4 (precursor *m/z* 263.023). Structural elucidation was based o the characteristic neutral loss of the SO_3 group of the sulphated metabolites (79.957 Da) and its hydrated form H_2SO_4 (97.967 Da). (c) Chemical structures assigned to the precursor (*m/z* 263.023) and the product ions acquired by the tandem MS. (d) Spatial correlation, considering all acquired spectra in both brain levels, between ions *m/z* 263.023 (annotated as MHPG-SO_4) and *m/z* 249.007. The correlation coefficient of 0.62 indicates high spatial correlation between the two ions advocating towards their involvement in similar metabolic pathways. (e) Tandem MS spectra (*m/z* values extracted at 1.5 signal-to-noise ratio) collected from rat brain tissue sections (15V) for metabolite identification of DOPEG-SO_4 (precursor *m/z* 249.007). (f) Chemical structures assigned to the precursor (*m/z* 240.007) and the product ions acquired by the tandem MS. (g) Tandem MS spectra (*m/z* values extracted at 1.5 signal-to-noise ratio) collected from rat brain tissue sections (10V) for metabolite identification of HVA-SO_4 (precursor *m/z* 261.007). (h) Chemical structures assigned to the precursor (*m/z* 261.008) and the product ions acquired by the tandem MS. (i) Spatial correlation, considering all acquired spectra in both brain levels, between ions *m/z* 261.008 (annotated as HVA-SO_4) and *m/z* 246.992 (annotated as DOPAC-SO_4). The correlation coefficient of 0.67 indicates high spatial correlation between the two ions advocating towards their involvement in similar metabolic pathways. On-tissue fragmentation was performed when sufficient precursor ion intensity was available. Precursor ions were isolated using a 1 Da window, followed by quadrupole selection and fragmentation in the collision cell. Collision energies were optimized for each analyte, ranged from 5.0 to 20 V. In each tandem mass spectrum, the product ion formed after the characteristic neutral loss of SO_3 is indicated with #. Data were collected in negative ionization mode using 9-AA as the MALDI matrix.

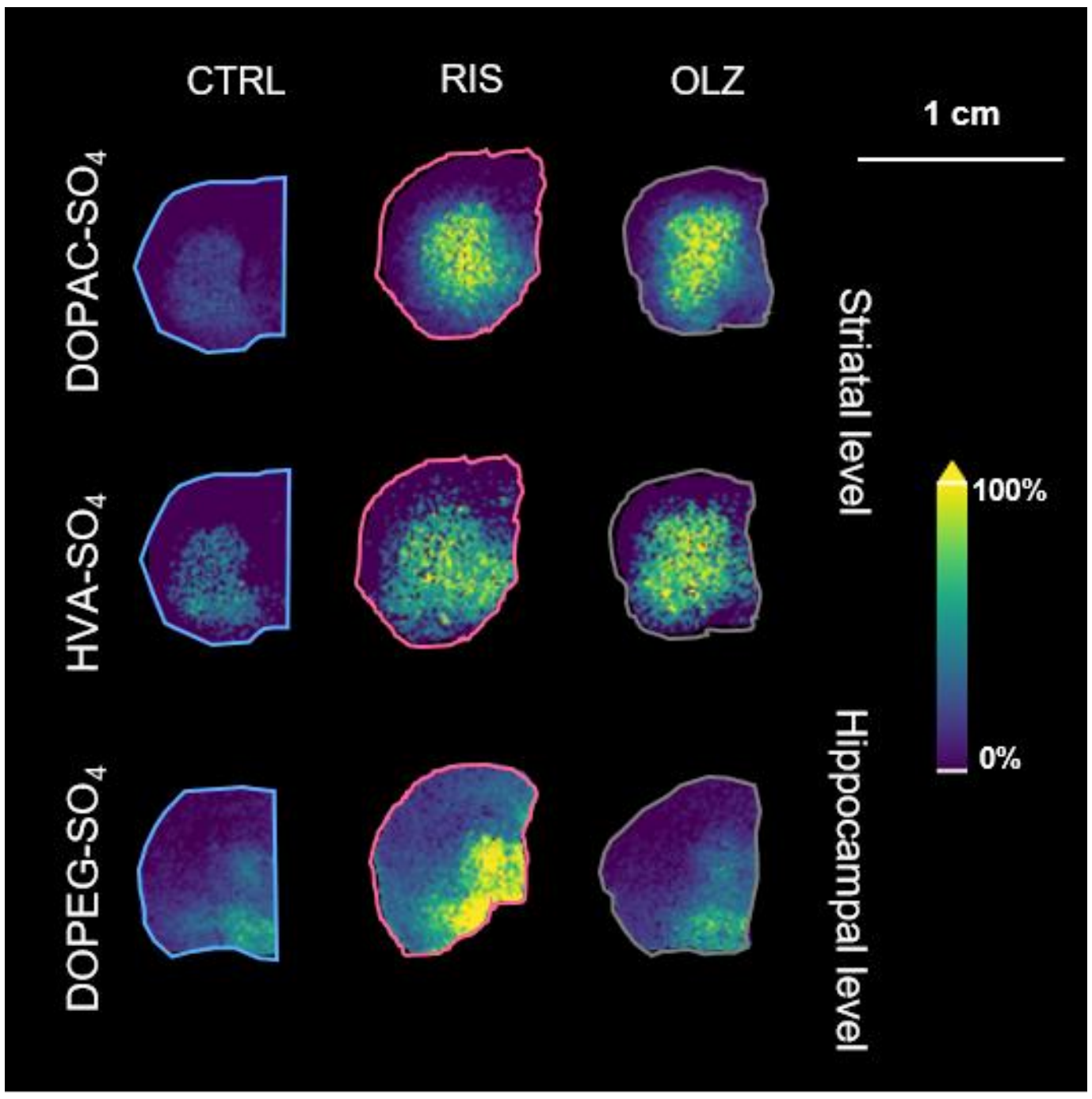

Figure S8. Region-specific alterations in sulphated catecholaminergic metabolites following RIS and OLZ treatment. Representative MALDI-MSI images of DOPAC-SO_4 (*m/z* 246.992), HVA-SO_4 (*m/z* 261.008) and DOPEG-SO_4 (*m/z* 249.007) in rat brain tissue sections across CTRL, RIS, and OLZ groups. Ion images were normalized using root mean square (RMS) and acquired at 100 µm spatial resolution. Data were collected in negative ionization mode using 9-AA as the MALDI matrix.

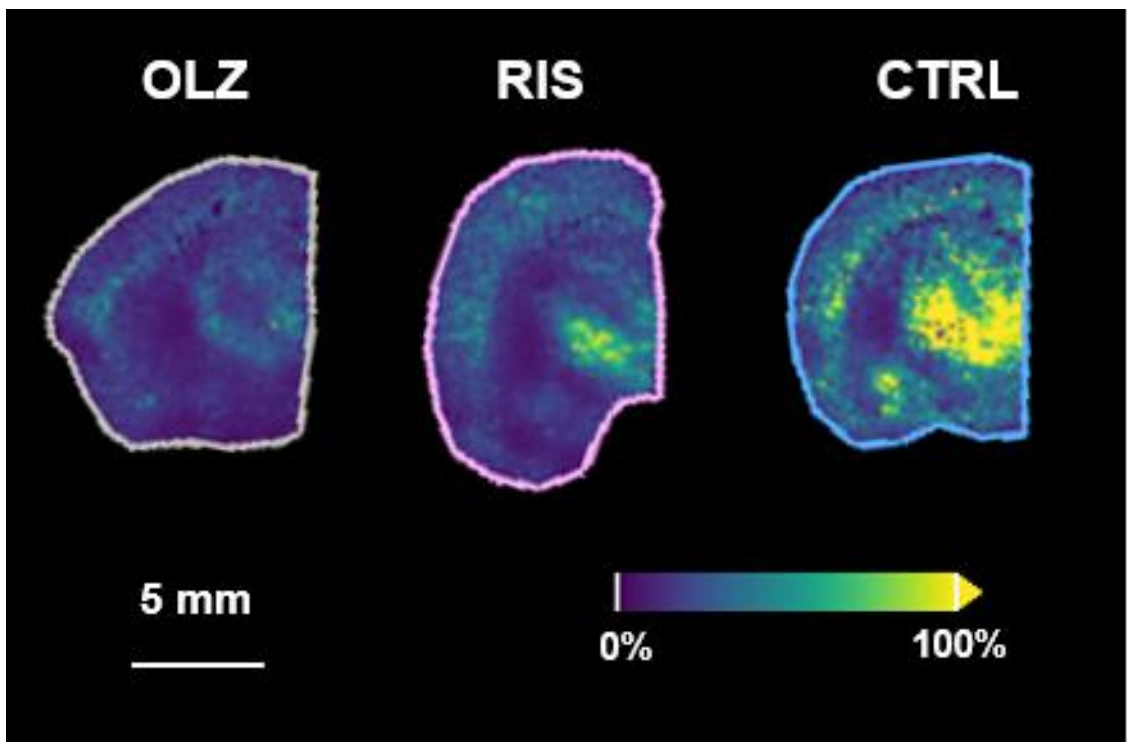

Figure S9. Serotonin (5-HT) and its metabolite 5-hydroxyindoleacetic acid (5-HIAA) in brain tissue following antipsychotic treatment. The ratio of 5-HIAA to 5-HT (5-HIAA/5-HT) was used to assess the apparent serotonin turnover. A trend toward decreased 5-HT metabolism was observed in the OLZ-treated group, suggesting subtle modulation of serotonergic activity. Data were collected in positive ionization mode using FMP-10 as reactive MALDI matrix.

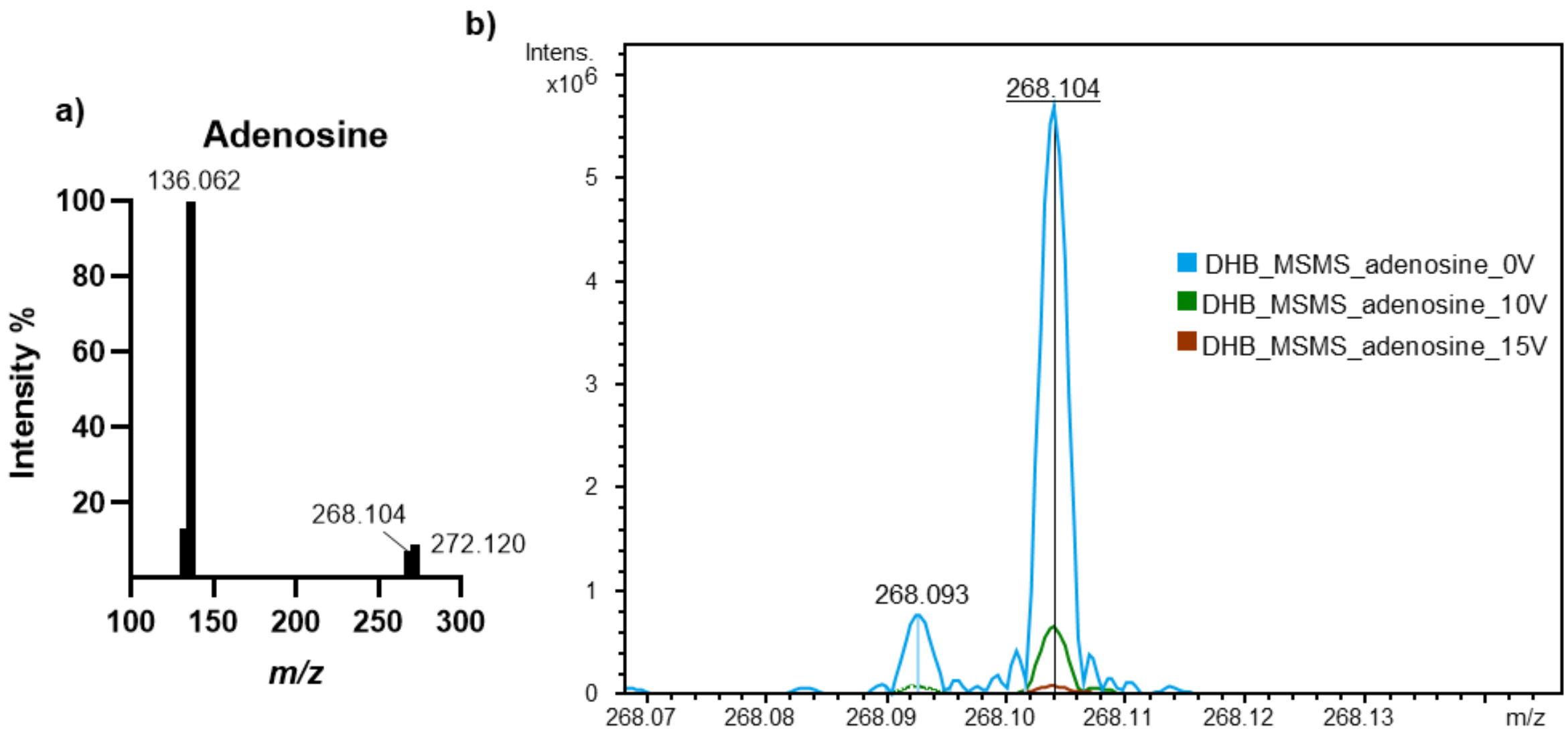

Figure S10. Structural validation of adenosine on rat brain tissue sections. (a) Tandem MS spectra (*m/z* values extracted at 4 signal-to-noise ratio) collected from rat brain tissue sections (10V) for metabolite identification of adenosine (precursor *m/z* 268.104). The spectrum was validated through comparison with the experimentally acquired tandem mass spectrum in MoNA data base (VF-NPL-QTOF006510) for adenosine. (b) Overlaid mass spectra collected with 0V (blue), 10V (green) and 15V (brown) showing the decay of the precursor peak (*m/z* 268.104) with increasing collision energy. Data were collected in positive ionization mode using DHB as the MALDI matrix.

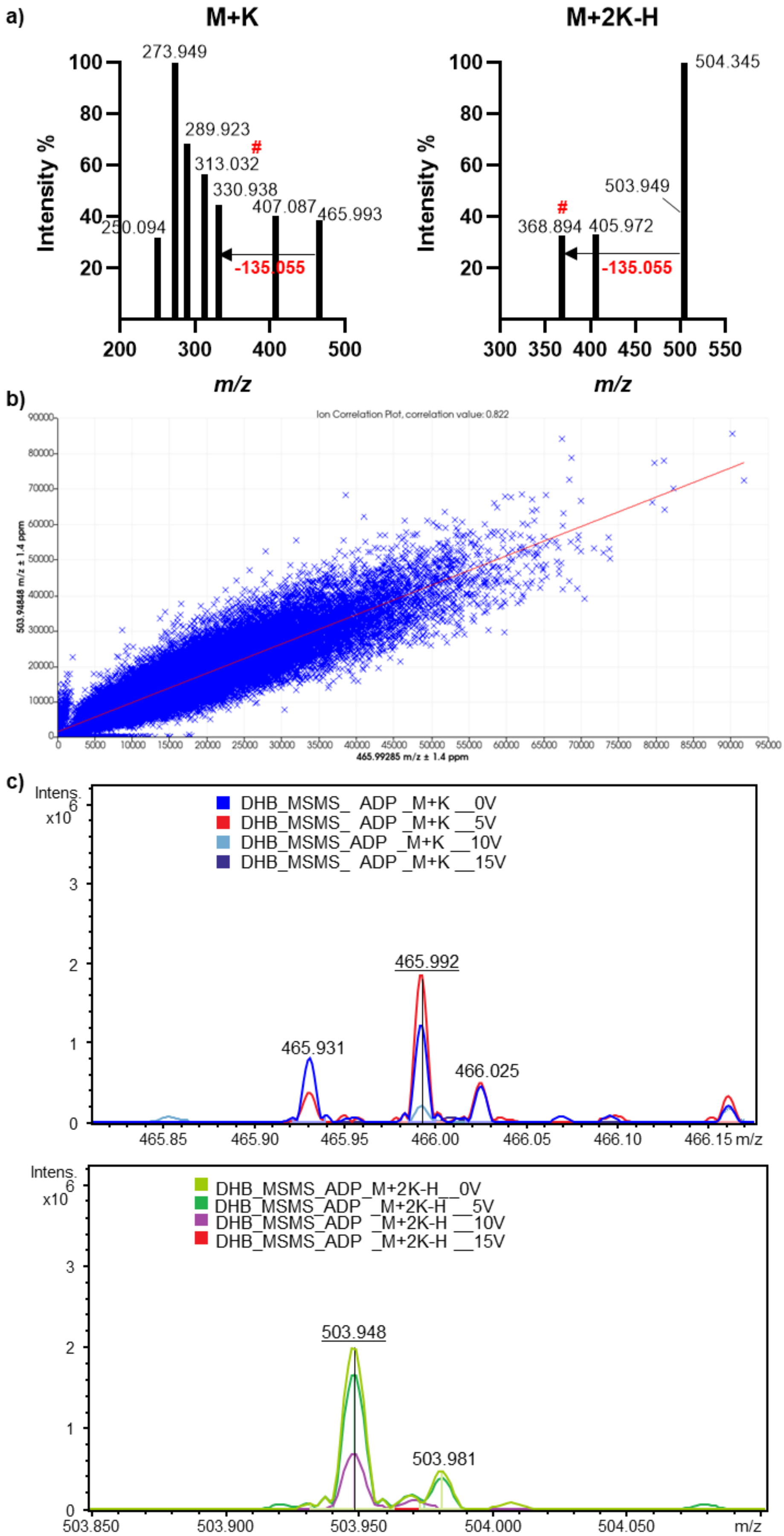
a)
M+K
M+2K-H
Intensity %
m/z
273.949
289.923
313.032
330.938
250.094
407.087
465.993
-135.055
#
504.345
503.949
368.894
405.972
-135.055
#
b)
Ion Correlation Plot, correlation value: 0.822
503.94848 m/z ± 1.4 ppm
465.99285 m/z ± 1.4 ppm
c)
Intens.
x10^6
DHB_MSMS_ ADP _M+K __0V
DHB_MSMS_ ADP _M+K __5V
DHB_MSMS_ADP _M+K __10V
DHB_MSMS_ ADP _M+K __15V
465.992
465.931
466.025
m/z
Intens.
x10^6
DHB_MSMS_ADP_M+2K-H__0V
DHB_MSMS_ADP _M+2K-H __5V
DHB_MSMS_ADP _M+2K-H __10V
DHB_MSMS_ADP _M+2K-H __15V
503.948
503.981
m/z

Figure S11. Structural validation of adenosine diphosphate on rat brain tissue sections. (a) Tandem MS spectra (*m/z* values extracted at 1.5 signal-to-noise ratio) collected from rat brain tissue sections (10V) for metabolite identification of ADP detected as two adducts, i.e., M+K (precursor *m/z* 465.993) and M+2K-H (precursor *m/z* 503.949). Structural elucidation was primarily based on the neutral loss of adenine (135.055 Da). (b) Spatial correlation, considering all acquired spectra in striatal brain level, between ions *m/z* 465.993 (annotated as ADP M+K) and *m/z* 503.949 (annotated as ADP M+2K-H). The correlation coefficient of 0.82 indicates very high spatial correlation between the two ions advocating towards their belonging on the same molecular species. (c) Overlaid mass spectra of ADP M+K (upper spectrum) and ADP M+2K-H (lower spectrum) collected with 0V-15V showing the decay of the precursor peak (*m/z* 465.993 and 503.949, respectively) with increasing collision energy. Data were collected in positive ionization mode using DHB as the MALDI matrix.

a)

	508.00314 m/z ± 1.4 ppm	545.95896 m/z ± 4.5 ppm	583.91463 m/z ± 2.3 ppm
508.00314 m/z ± 1.4 ppm	1	0.707	0.535
545.95896 m/z ± 4.5 ppm	0.707	1	0.652
583.91463 m/z ± 2.3 ppm	0.535	0.652	1

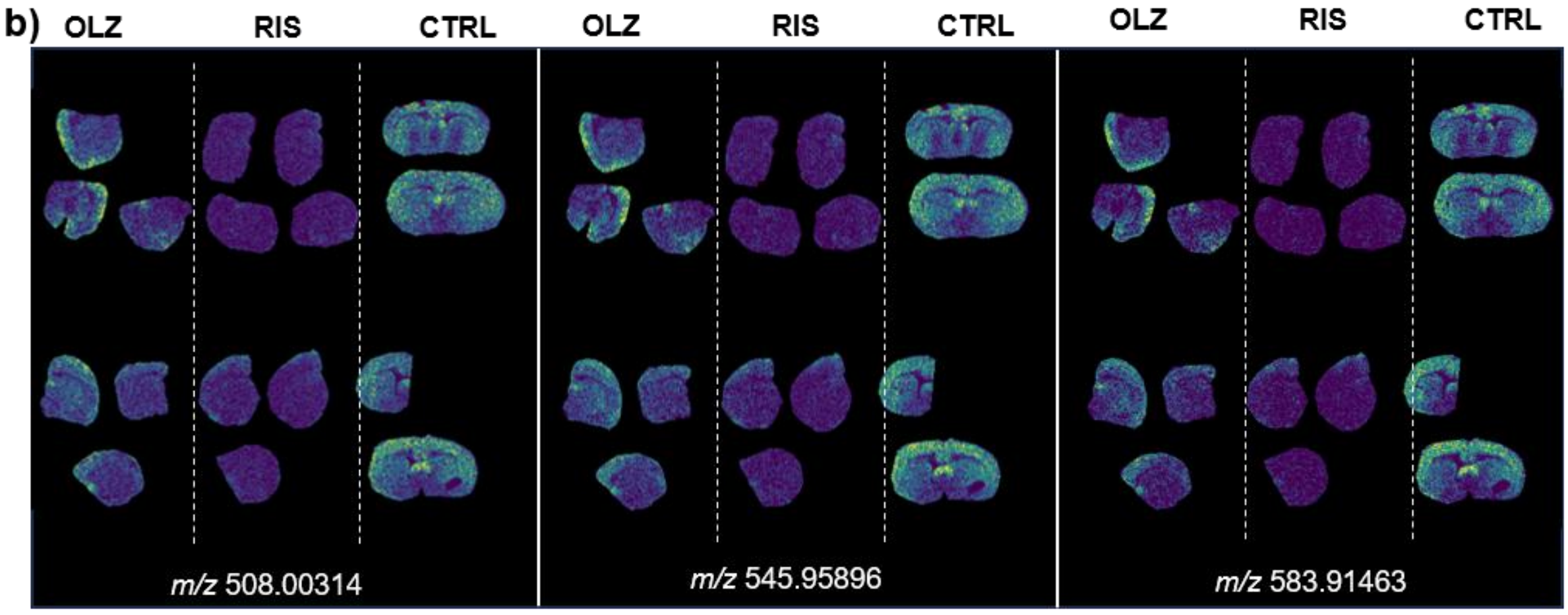

Figure S12. Structural validation of adenosine triphosphate (ATP) on rat brain tissue sections. (a) Correlation matrix of the spatial correlation among the detected ATP adducts on coronal rat brain tissue sections at the striatal level using DHB as matrix. (b) Corresponding MALDI-MS ion images of the same species (data normalized to RMS). *m/z* 508.003, M+H; *m/z* 545.959, M+K; *m/z* 583.914, M+2K-H.

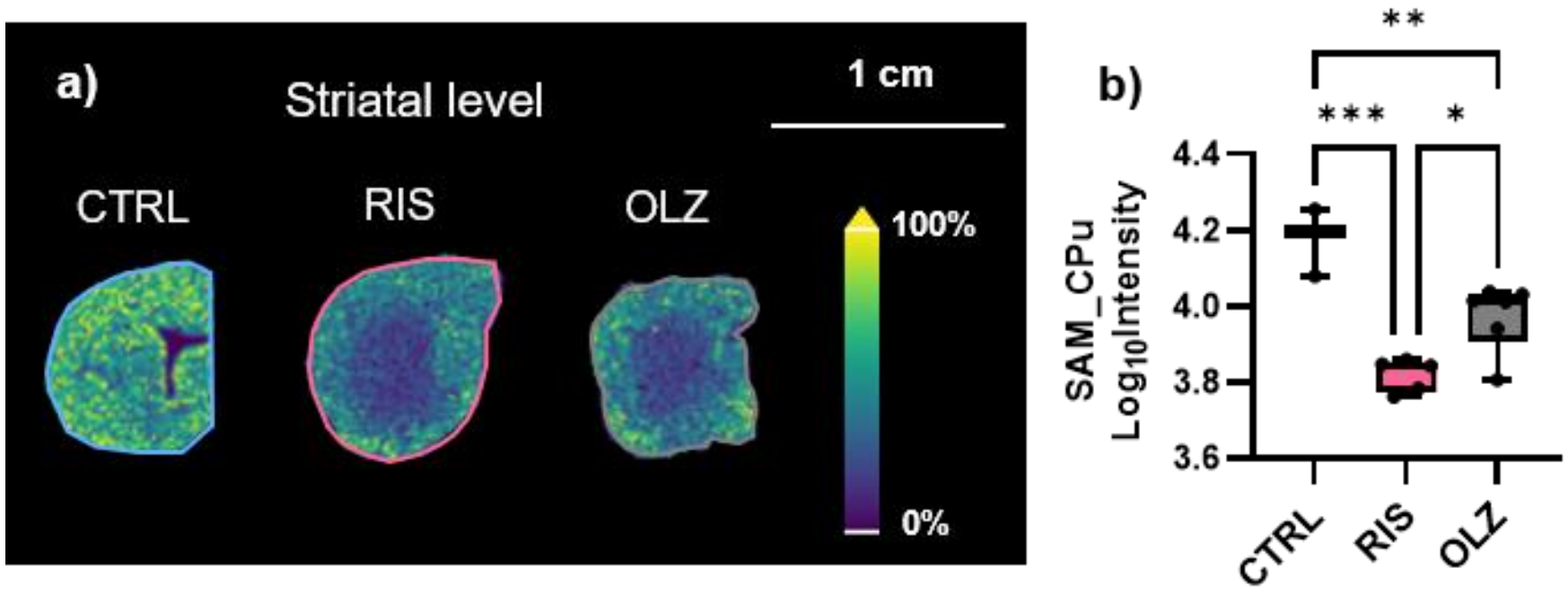

Figure S13. Region-specific alterations in S-adenosyl methionine (SAM) levels following RIS and OLZ treatment. (a) Representative MALDI-MSI images of SAM (*m/z* 399.144) in rat brain tissue sections across CTRL, RIS, and OLZ groups. Ion images were normalized using root mean square (RMS) and acquired at 100 µm spatial resolution. (a) Representative box plot showing log_{10}-normalized intensity of SAM in the CPu. Box plots indicate statistically significant group differences, presented by asterisks: * $p \leq 0.05$, and ** $p \leq 0.01$, ***$p \leq 0.001$, ****$p \leq 0.0001$. Data were collected in positive ionization mode using DHB as the MALDI matrix.

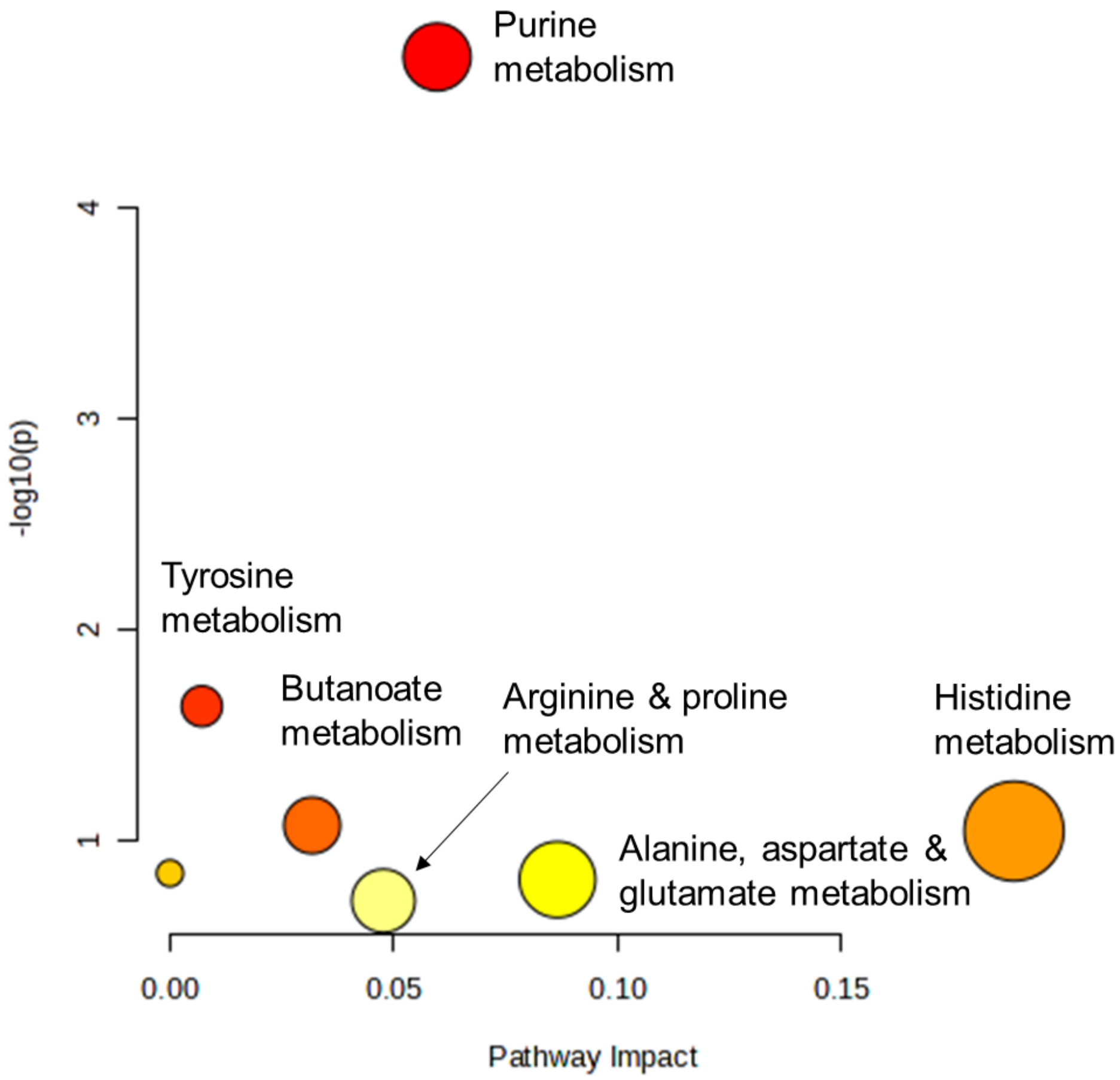

Pathway Name	Match Status	p	-log(p)	Holm p	FDR	Impact	Details
Purine metabolism	5/71	1.928E-5	4.7149	0.0015424	0.0015424	0.05966	KEGG
Tyrosine metabolism	2/42	0.022933	1.6395	1.0	0.91731	0.00712	KEGG
Butanoate metabolism	1/15	0.084114	1.0751	1.0	1.0	0.03175	KEGG
Histidine metabolism	1/16	0.089491	1.0482	1.0	1.0	0.18852	KEGG
One carbon pool by folate	1/26	0.14174	0.84851	1.0	1.0	0.0	KEGG
Alanine, aspartate and glutamate metabolism	1/28	0.15186	0.81855	1.0	1.0	0.08654	KEGG
Arginine and proline metabolism	1/36	0.1913	0.71828	1.0	1.0	0.04767	KEGG

Figure S14. KEGG pathway enrichment analysis of differentially affected metabolites following antipsychotic treatment. Pathway enrichment was performed based on the Kyoto Encyclopedia of Genes and Genomes (KEGG), restricted to *Rattus norvegicus*. The *x*-axis represents the statistical significance of enrichment as -$\log_{10}$(*p*-value), where values >1.3 correspond to $p < 0.05$. The *y*-axis displays pathway impact values, indicating the relative importance of each pathway based on topology analysis. Circle size denotes the enrichment ratio, with larger circles indicating higher ratios.

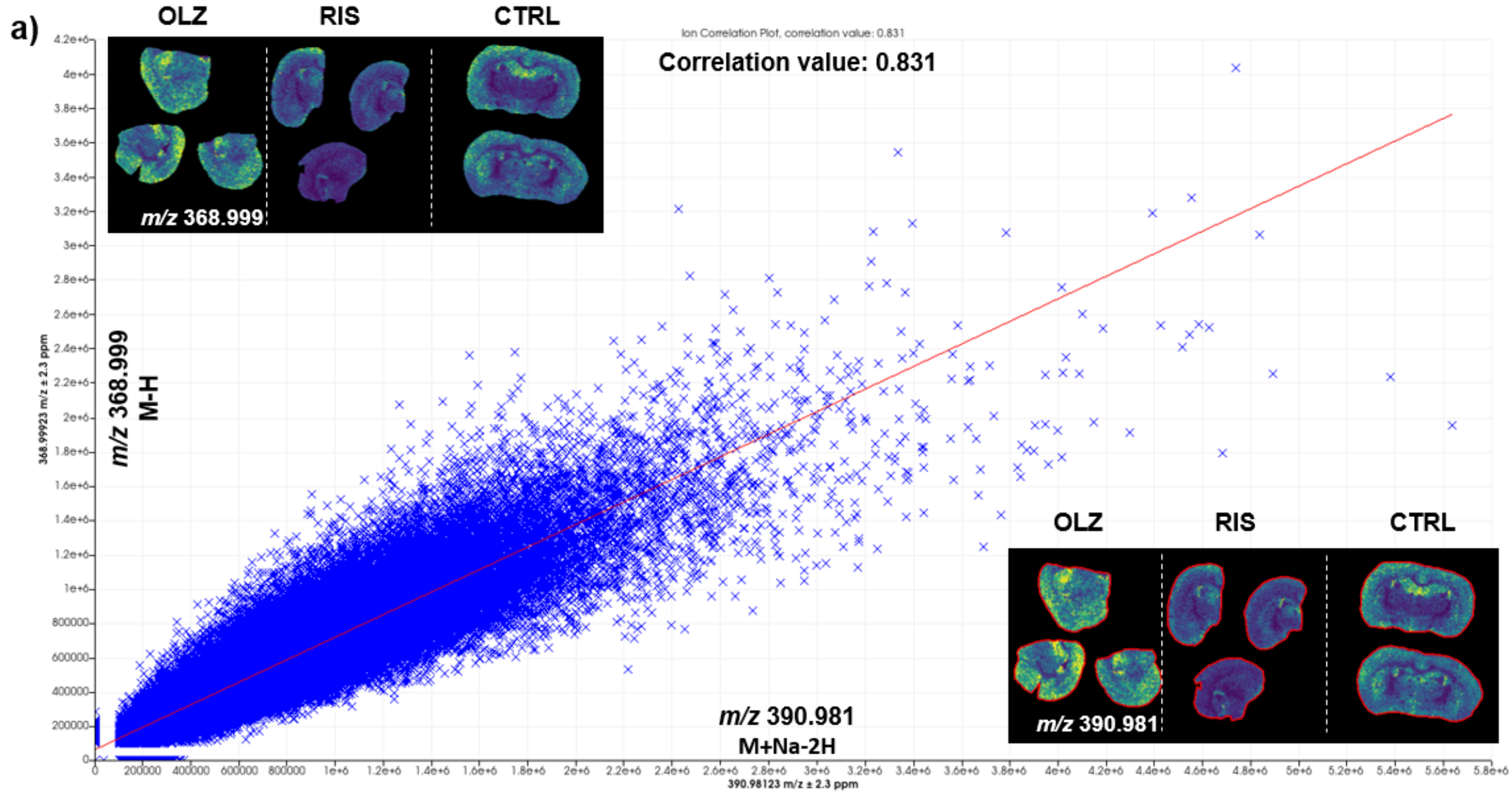

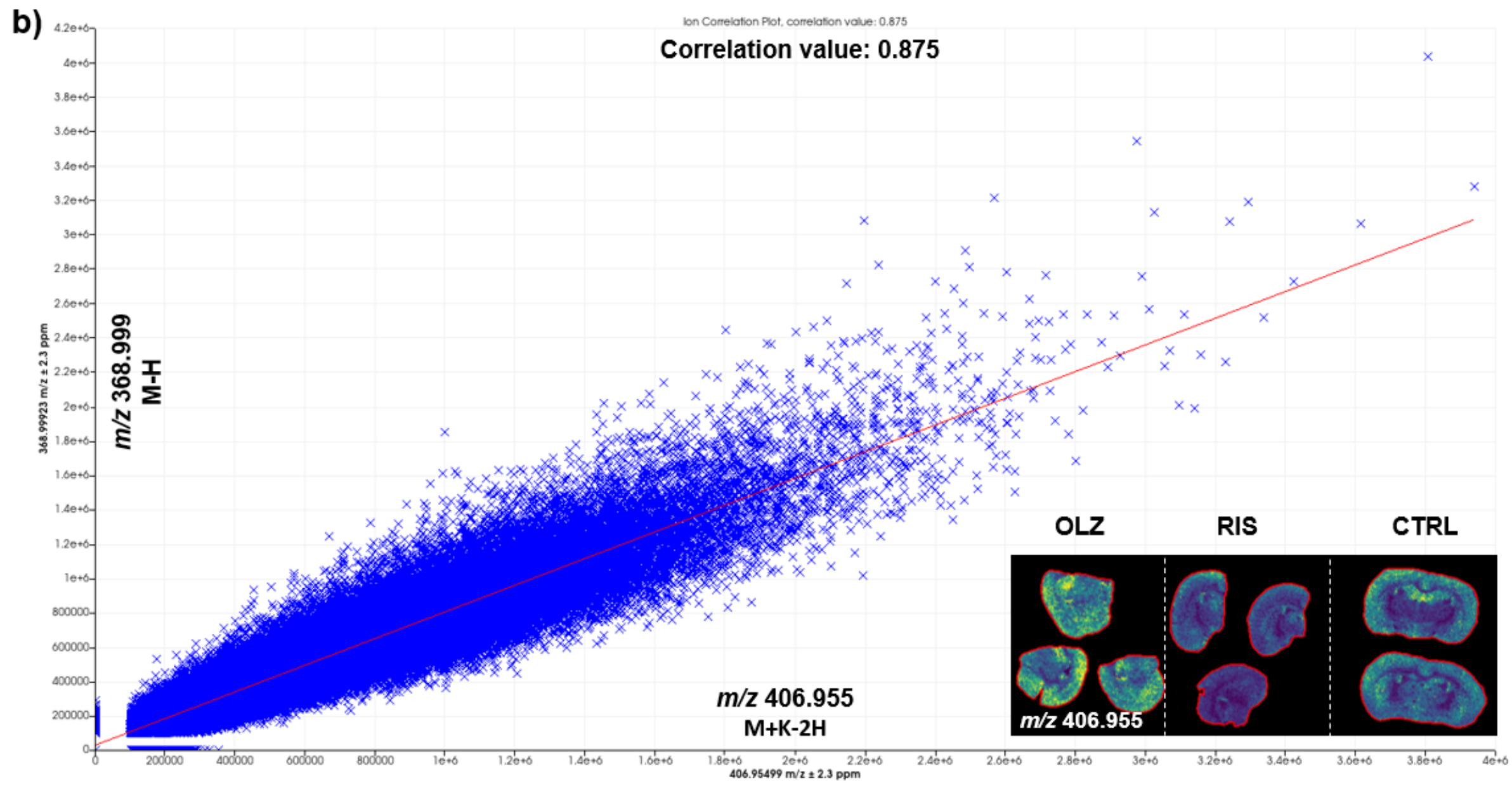

Figure S15. Structural validation of sedoheptulose biphosphate (SBP) using spatial correlation among multiple adducts in negative mode MALDI-MSI. (a) Spatial correlation, considering all acquired spectra in striatal and hippocampal brain levels, between ions *m/z* 368.999 (annotated as SBP M-H) and *m/z* 390.981 (annotated as SBP M+Na-2H). The correlation coefficient of 0.83 indicates very high spatial correlation between the two ions advocating towards their belonging on the same molecular species. Representative ion images on hippocampal level visually confirm the correlation. (b) Spatial correlation, considering all acquired spectra in striatal and hippocampal brain levels, between ions *m/z* 368.999 (annotated as SBP M-H) and *m/z* 406.955 (annotated as SBP M+K-2H). The correlation coefficient of 0.87 indicates very high spatial correlation between the two ions advocating towards their belonging on the same molecular species. Representative ion images on hippocampal level visually confirm the correlation.

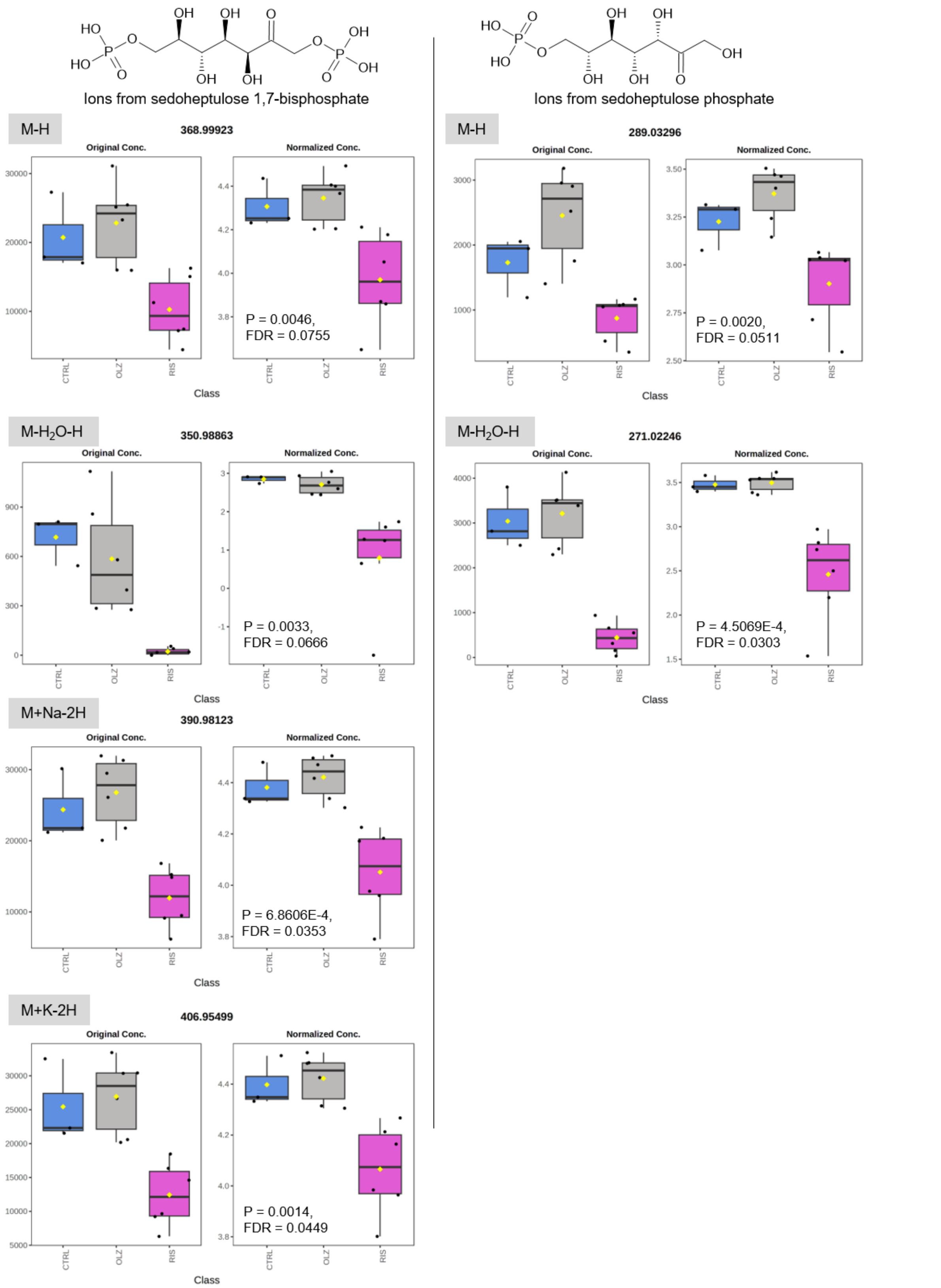

Figure S16. Structural validation of sedoheptulose biphosphate (SBP) by comparing treatment effects on multiple adducts o SBP and sedoheptulose phosphate. Sedoheptulose phosphate serves as identification tool for the structural validation of SBP since either it is endogenously localized in the brain or produced by in-source fragmentation demonstrates the same treatment effects as the precursor molecule.

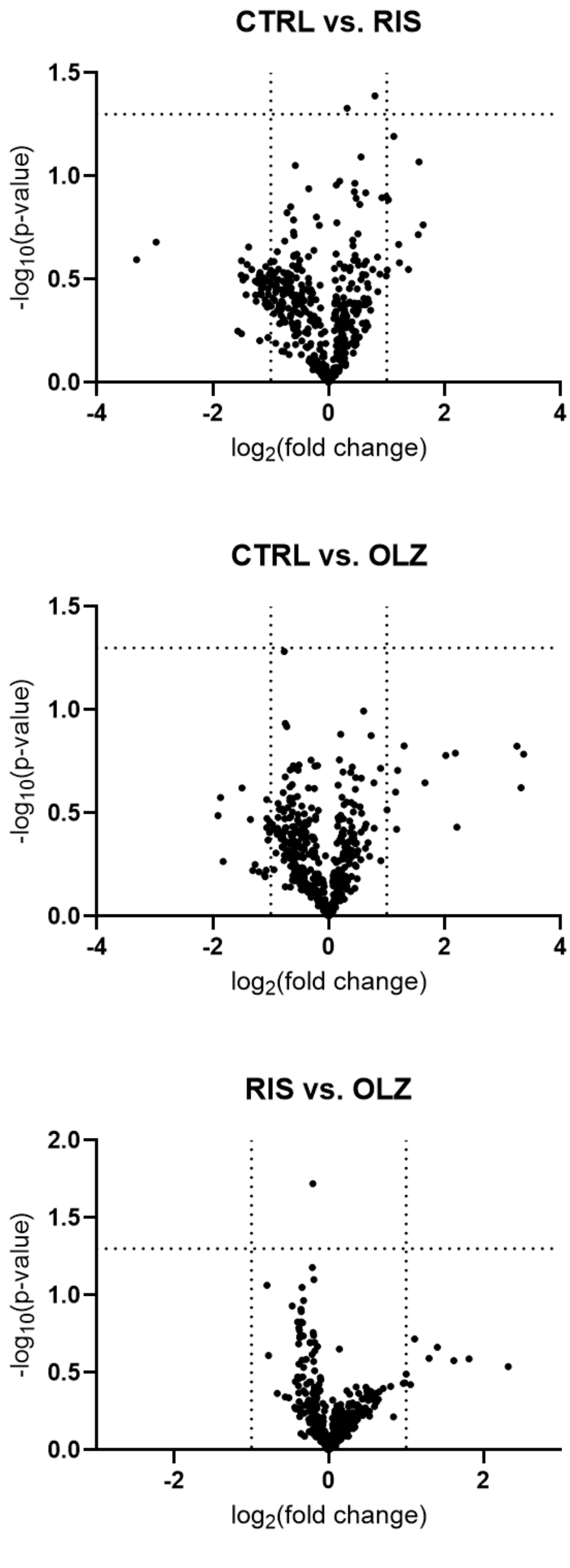

Figure S17. Volcano plots showing peptide-level comparisons between groups. No peptides met the significance threshold across comparisons, with all values falling below statistical and fold-change cut-offs. Significance criteria were defined as $p < 0.05$ (equivalent to 1.3010 on the $-\log_{10}$ scale) and fold change >2 (±1 on the $\log_2$ scale).

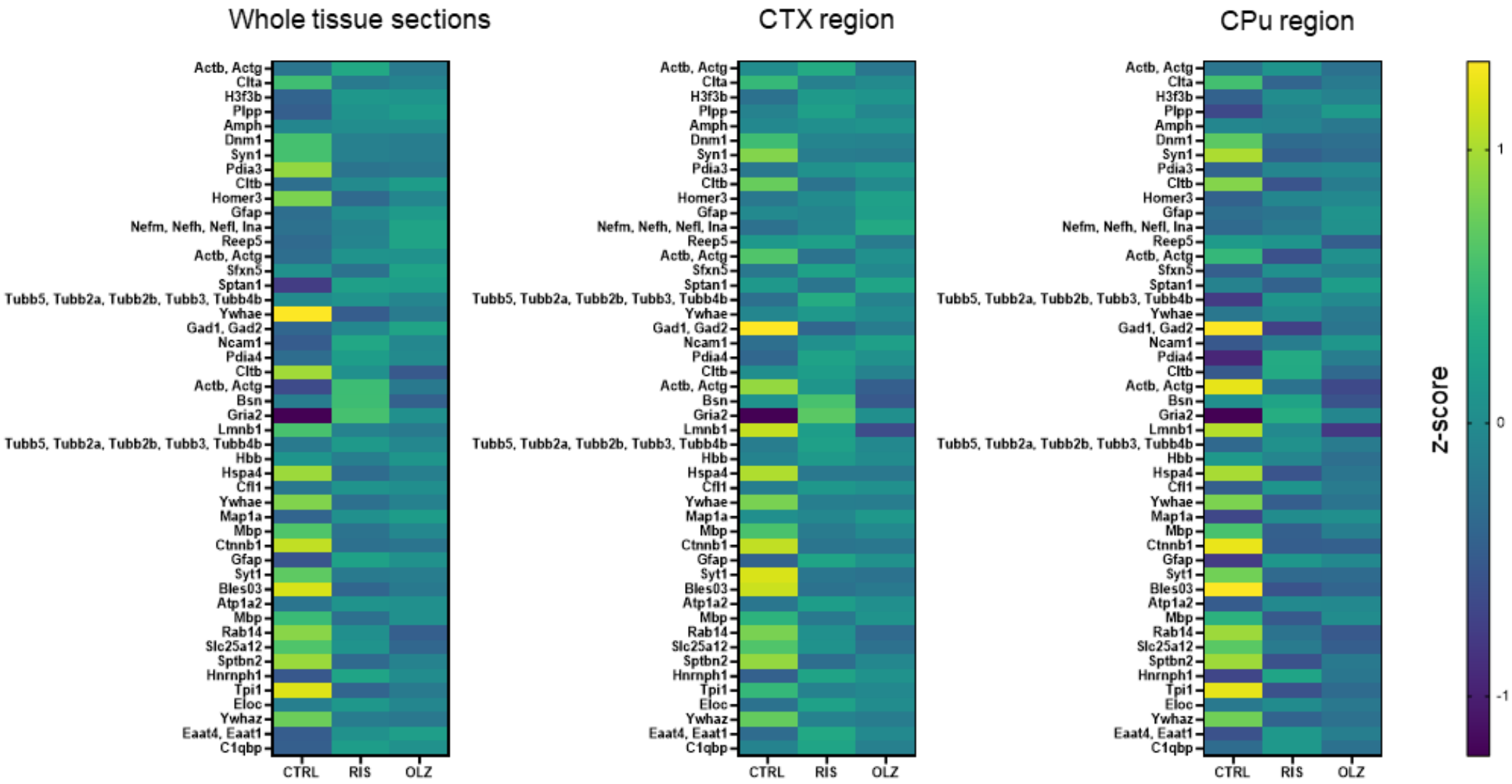

Figure S18. Z-score heat maps of mass-matched peptides across tissue sections and regions of interest (CTX and CPu) for group comparisons. Each row represents the scaled Z-score expression of a specific peptide feature, visualized using a yellow–dark blue color scale, where yellow indicates higher abundance and dark blue indicates lower abundance. Heat maps are shown for both whole tissue sections and specific regions of interest (CTX and CPu). Genes corresponding to each *m/z* value are listed in ascending order based on *m/z*.

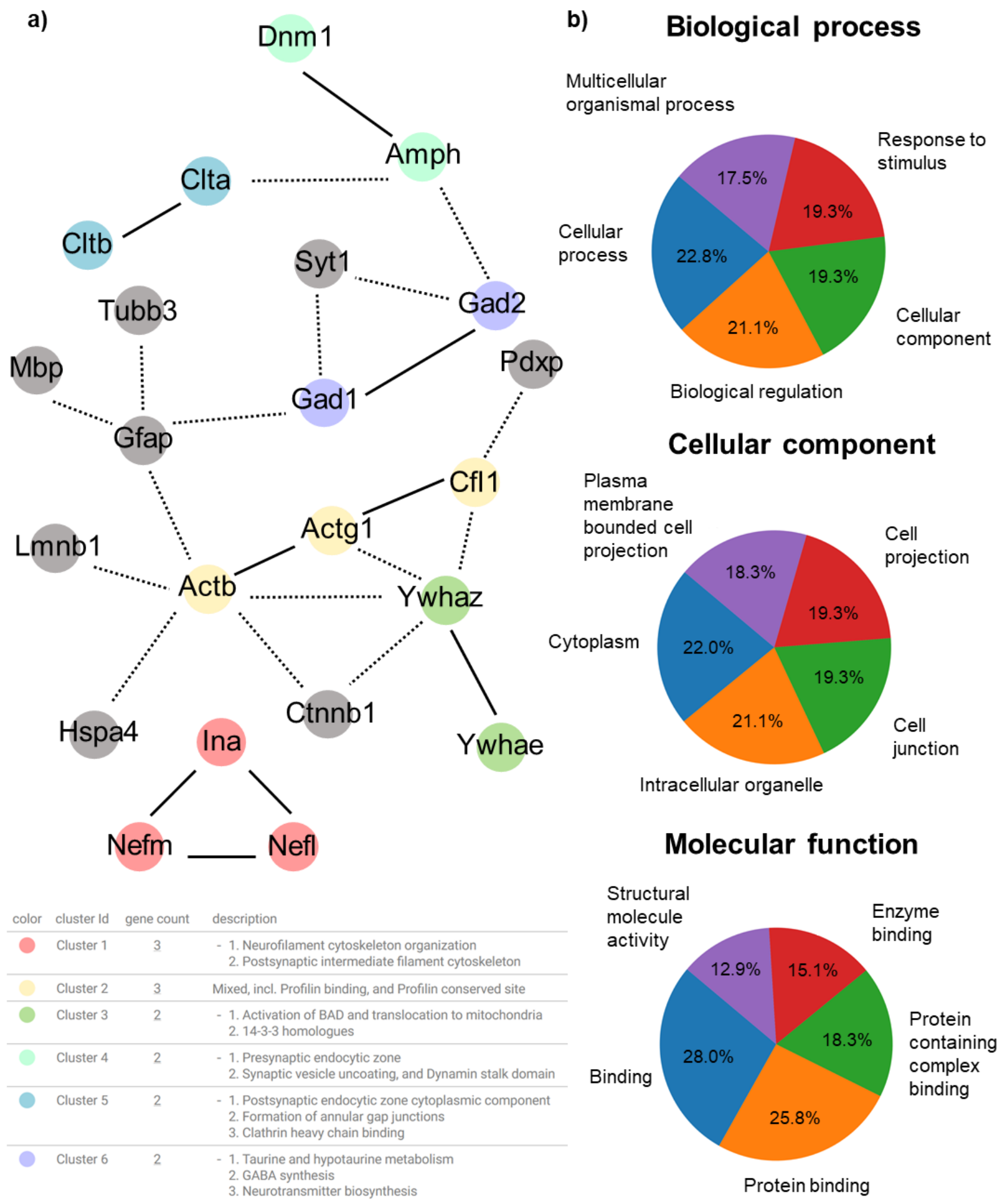

color	cluster Id	gene count	description
	Cluster 1	3	- 1. Neurofilament cytoskeleton organization<br>2. Postsynaptic intermediate filament cytoskeleton
	Cluster 2	3	Mixed, incl. Profilin binding, and Profilin conserved site
	Cluster 3	2	- 1. Activation of BAD and translocation to mitochondria<br>2. 14-3-3 homologues
	Cluster 4	2	- 1. Presynaptic endocytic zone<br>2. Synaptic vesicle uncoating, and Dynamin stalk domain
	Cluster 5	2	- 1. Postsynaptic endocytic zone cytoplasmic component<br>2. Formation of annular gap junctions<br>3. Clathrin heavy chain binding
	Cluster 6	2	- 1. Taurine and hypotaurine metabolism<br>2. GABA synthesis<br>3. Neurotransmitter biosynthesis

Figure S19. Functional interaction and pathway analysis of antipsychotic drug effects. (a) Protein-protein interaction (PPI) network illustrating functional and physical associations among proteins identified in RIS- and OLZ-treated brains. Edges represent interactions, with line thickness indicating the strength of supporting evidence. DBSCAN clustering algorithm revealed six distinct protein clusters, each associated with specific biological functions such as cytoskeletal organization, synaptic endocytosis, mitochondrial function, and taurine metabolism. (b) Gene Ontology (GO) enrichment analysis highlighting the top five functional categories of the observed proteins, classified into biological processes, cellular components, and molecular functions.

References

1. Shariatgorji, M., et al., *Comprehensive mapping of neurotransmitter networks by MALDI-MS imaging.* Nat Methods, 2019. **16**(10): p. 1021-1028.
2. Vallianatou, T., et al., *Derivatization of Small Molecules using Deuterated Analog of Reactive Matrix to Facilitate Identification in Mass Spectrometry Imaging.* Chemistry–Methods, 2025. **5**(12): p. e202500062.
3. Fridjonsdottir, E., et al., *Region-Specific and Age-Dependent Multitarget Effects of Acetylcholinesterase Inhibitor Tacrine on Comprehensive Neurotransmitter Systems.* ACS Chem Biol, 2022. **17**(1): p. 147-158.
4. Vallianatou, T., et al., *Rapid Metabolic Profiling of 1 µL Crude Cerebrospinal Fluid by Matrix-Assisted Laser Desorption/Ionization Mass Spectrometry Imaging Can Differentiate.* Anal Chem, 2023. **95**(50): p. 18352-18360.
5. Shariatgorji, M., et al., *Comprehensive mapping of neurotransmitter networks by MALDI–MS imaging.* Nat Methods, 2019. **16**(10): p. 1021-1028.
6. Vallianatou, T., et al., *Rapid Metabolic Profiling of 1 µL Crude Cerebrospinal Fluid by Matrix-Assisted Laser Desorption/Ionization Mass Spectrometry Imaging Can Differentiate De Novo Parkinson's Disease.* Anal Chem, 2023. **95**(50): p. 18352-18360.
7. Fridjonsdottir, E., et al., *Mass spectrometry imaging identifies abnormally elevated brain l-DOPA levels and extrastriatal monoaminergic dysregulation in l-DOPA-induced dyskinesia.* Sci Adv, 2021. **7**(2): p. eabe5948.
8. Enomoto, H., S. Takeda, and H. Hatta, *Spatial Analysis of Phosphatidylinositol Molecular Species in Pork Chop Tissues Using Matrix-assisted Laser Desorption/ionization-Mass Spectrometry Imaging.* J Oleo Sci, 2021. **70**(7): p. 979-987.
9. Ridenour, W.B., et al., *Structural Characterization of Phospholipids and Peptides Directly from Tissue Sections by MALDI Traveling-Wave Ion Mobility-Mass Spectrometry.* Anal Chem, 2010. **82**(5): p. 1881-1889.
10. Høiem, T.S., et al., *An optimized MALDI MSI protocol for spatial detection of tryptic peptides in fresh frozen prostate tissue.* Proteomics, 2022. **22**(10): p. e2100223.
11. Zubair, F., et al., *Trypsin and MALDI matrix pre-coated targets simplify sample preparation for mapping proteomic distributions within biological tissues by imaging mass spectrometry.* J Mass Spectrom, 2016. **51**(12): p. 1168-1179.
12. Heijs, B., et al., *Comprehensive Analysis of the Mouse Brain Proteome Sampled in Mass Spectrometry Imaging.* Anal Chem, 2015. **87**(3): p. 1867-1875.
